# mRNA-LNP prime–boost evolves precursors toward VRC01-like broadly neutralizing antibodies in preclinical humanized mouse models

**DOI:** 10.1126/sciimmunol.adn0622

**Published:** 2024-05-16

**Authors:** Xuesong Wang, Christopher A. Cottrell, Xiaozhen Hu, Rashmi Ray, Maria Bottermann, Paula Maldonado Villavicencio, Yu Yan, Zhenfei Xie, John E. Warner, Jordan Renae Ellis-Pugh, Oleksandr Kalyuzhniy, Alessia Liguori, Jordan R. Willis, Sergey Menis, Sebastian Rämisch, Saman Eskandarzadeh, Michael Kubitz, Ryan Tingle, Nicole Phelps, Bettina Groschel, Sunny Himansu, Andrea Carfi, Kathrin H. Kirsch, Stephanie R. Weldon, Usha Nair, William R. Schief, Facundo D. Batista

**Affiliations:** 1 The Ragon Institute of Mass General, MIT, and Harvard; Cambridge, 02139, USA.; 2 Department of Immunology and Microbiology, The Scripps Research Institute; La Jolla, 92037, USA.; 3 IAVI Neutralizing Antibody Center, The Scripps Research Institute; La Jolla, 92037, USA.; 4 Center for HIV/AIDS Vaccine Immunology and Immunogen Discovery, The Scripps Research Institute; La Jolla, 92037, USA.; 5 Moderna Inc.; Cambridge, 02139, USA.; 6 Department of Biology, Massachusetts Institute of Technology; Cambridge, 02139, USA.

**Keywords:** VRC01, germline-targeting immunogen, HIV, mRNA-LNP vaccine, broadly neutralizing antibody, somatic hypermutation

## Abstract

Germline-targeting (GT) protein immunogens to induce VRC01-class broadly neutralizing antibodies (bnAbs) to the CD4 binding site (CD4bs) of the HIV Envelope have shown promise in clinical trials. Here, we preclinically validated a lipid nanoparticle (LNP)–encapsulated nucleoside mRNA (mRNA-LNP) encoding eOD-GT8 as a soluble self-assembling 60mer nanoparticle in mouse models. In a model with three humanized B cell lineages bearing distinct VRC01-precursor B cell receptors (BCRs) with similar affinities for eOD-GT8, all lineages could be simultaneously primed and undergo diversification and affinity maturation without exclusionary competition. Boosts drove precursor B cell participation in germinal centers, the accumulation of somatic hypermutations, including in key VRC01-class positions, and affinity maturation to boost and native-like antigens in two of the three precursor lineages. We have preclinically validated a prime–boost regimen of soluble self-assembling nanoparticles encoded by mRNA-LNP, demonstrating that multiple lineages can be primed, boosted, and diversified along the bnAb pathway.

## One-Sentence Summary

mRNA-LNP prime and boost immunogens can drive the maturation of multiple VRC01 precursors in the same host.

## INTRODUCTION

The isolation of broadly neutralizing antibodies (bnAbs) to conserved sites on the HIV envelope glycoprotein (Env) protein has reinvigorated vaccine development (1). Though bnAbs isolated from patients recognize HIV with high affinity, bnAb precursors mainly do not (2–5). A promising approach to overcome this difficulty is germline targeting (GT) vaccination, wherein an initial prime activates bnAb precursors and recruits these precursors to germinal centers (GCs), after which increasingly Env-like boost immunogens would drive line expansion and increased affinity for native Env (3, 6–10).

Three GT immunogens undergoing clinical trials (426c.Mod.Core-C4b: NCT05471076; BG505 SOSIP.GT1.1 gp140: NCT04224701; and eOD-GT8 60mer: NCT03547245 (11)) are designed around VRC01-class bnAbs targeting the Env CD4-binding site (CD4bs) (12–15). Mice expressing human antibodies have been key to preclinical testing of GT vaccine candidates (6, 8, 10, 16–24), including for CD4bs (6, 17–19, 21, 24). We recently developed a CRISPR/Cas9 method to generate mice bearing human B cell receptors (BCR) (25, 26). This approach yielded the CLK series of mice, which bear prerearranged VDJ regions from genuine, non-inferred precursors from the VRC01 subclasses N6 (which uses Vκ1–33) (27) and PCIN63 (Vκ1–5) (28, 29). CLK B cells could be primed by eOD-GT8 60mer protein and undergo VRC01-like somatic hypermutation (SHM) even when titrated to low, physiologically relevant precursor frequencies (26). Boost-stage applications would therefore be a logical extension for these models.

Boost immunogens must drive the evolution of key mutations necessary for mature-like breadth and potency (6–8, 10, 21, 30–32). However, shepherding bnAb development is challenging (6, 7, 33) and requires the accumulation of often improbable SHMs (34). Immunogens must select for and then guide the subset of naïve precursors that have accumulated either functional SHMs or useful intermediate mutations (31).

Another potential complication is posed by B cell competition and immunodominance, which are affected by affinity to antigen, precursor frequency, antigen avidity, and other factors (35, 36). Precursors to bnAbs may be outcompeted in GCs by higher-affinity clones lacking the capacity for later development into bnAbs (37). The nature of boost-phase competition is somewhat unclear; secondary GCs are primarily populated by naive B cells, but some memory B cells (MBCs) can be recruited (38–40), though the factors allowing reentry are disputed (41–43). Instead of driving new reactions, boosting may also increase T cell help in ongoing GCs (44). A greater understanding of B cell immunodominance in GCs after priming and boosting would consequently facilitate vaccine design.

The role of lipid nanoparticle (LNP)–encapsulated nucleoside mRNA vaccines (mRNA-LNP) in combating the SARS-CoV-2 pandemic (45, 46) has rendered them an attractive format for other infectious diseases. mRNA vaccines may overcome some challenges associated with GT: immunogens may be produced for up to 10 days post-administration (47), which may allow precursors to remain longer in GCs and undergo further SHM. Furthermore, mRNA-LNP can encode complex nanoparticle immunogens and elicit B cell expansion in mouse models (48).

Approved mRNA-LNP vaccines express membrane-bound proteins (49, 50). Here, by contrast, we used knock-in (KI) mouse models to investigate soluble self-assembling nanoparticles encoded by mRNA-LNP. We found that eOD-GT8 60mer mRNA-LNP can not only prime an array of true human germline sequences at low frequencies but can also prime multiple precursor B cell lineages simultaneously in the same host. We then validated three mRNA-LNP boost nanoparticles, including one currently undergoing clinical trial (NCT05001373). All boosters enhanced the secondary GC response and drove affinity maturation in two of these lineages, allowing them to recognize more native-like HIV Env. Thus, this soluble nanoparticle prime–boost regimen encoded by mRNA-LNP triggers the evolution of VRC01 bnAb precursors in humanized mouse models, laying the groundwork for further GT vaccine development.

## RESULTS

### eOD-GT8 60mer mRNA-LNP induces and maintains robust B cell responses

**Fig. 1.**
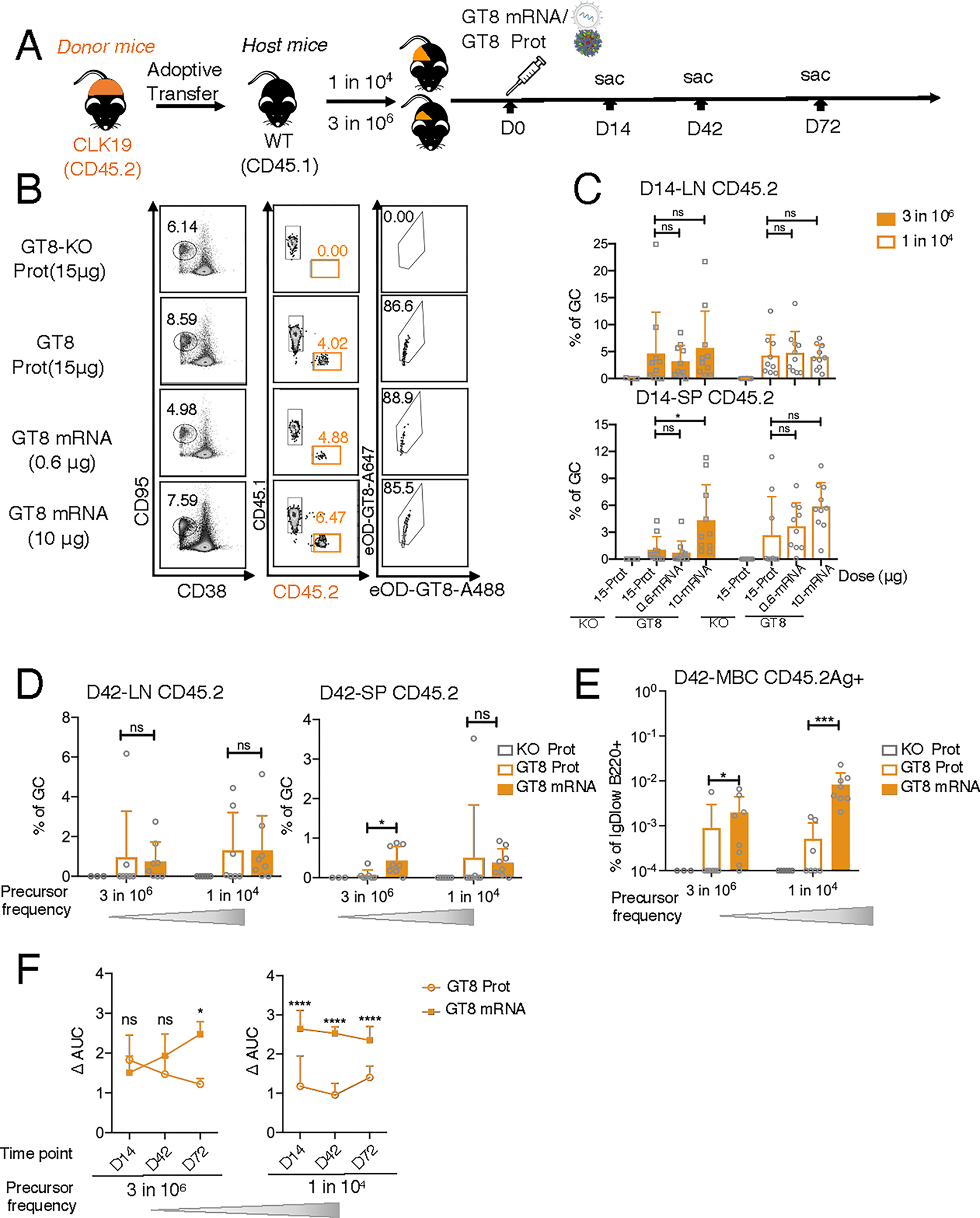
CLK19 B cell responses induced by eOD-GT8 60mer mRNA-LNP. (**A**) Schematic: 8-week-old WT CD45.1 mice adoptively transferred with B cells from CLK19 CD45.2 mice to establish precursor frequencies of ~1 in 10^4^ or 3 in 10^6^ B cells. Mice were then immunized with eOD-GT8 60mer formulated with alum (15 μg), a CD4bs-knockout control (eOD-GT8-KO 60mer; 15 μg), or eOD-GT8 60mer mRNA-LNP (0.6 or 10 μg) 1 day post transfer (D0). Splenocytes or lymph nodes (LNs) were isolated on D14, D42, and D72. “mRNA” is used to mean “mRNA-LNP throughout, and “prot” for “protein.” (**B**) Flow cytometry of D14 LN from mice (1 in 10^4^) as per (A). CD45.2-binder populations gated as Scatter/Singlet/Live (SSL^+^)/B220^+^/CD95^+^CD38^−^/CD45.2^+^/Ag^+^. GC percentage (first column), CD45.2 in GC (second), and Ag^+^ CD45.2 (third). (**C**) D14 LN (upper) or spleen (lower) quantification. The *x*-axis represents immunization groups and *y*-axis the percent CD45.2 within GC. Circles represent one mouse. Two independent experiments were pooled for analysis. n=3–5 mice per independent group. (**D**) D42 quantification. The *x*-axis indicates treatment group and the *y*-axis the percentage of CD45.2 within GCs. Two independent experiments were pooled for analysis. n=3–4 mice per independent group. (**E**) Ag^+^ CD45.2 MBC frequencies from D42 splenocytes. The gating strategy is shown in fig. S1E. *x*-axis shows immunization group precursor frequencies and *y*-axis represents the percentage of Ag^+^ CD45.2 MBC among the B220^+^IgD^lo^ GC population and starts at 0.0001. Two independent experiments were pooled for analysis; n=3–4 mice per independent group. Circles represent individual mice. (**F**) IgG titers from sera post immunization by eOD-GT8 60mer protein (15 μg) or mRNA-LNP (10 μg). n=3–10 mice in each group from two independent experiments. The *x*-axis indicates the day and the *y*-axis the change of area under curve (AUC_coated_ eOD_-GT8_−AUC_coated eOD-GT8 KO_). Where shown, bars indicate geometric means and geometric SD of pooled groups and significance was calculated with Student’s *t* test (D–F) or one-way ANOVA (C): *P*>0.05 represents no significance (ns), *P<0.05, ***P<0.001, and ****P<0.0001.

We previously found that an eOD-GT8 60mer protein prime can recruit B cells expressing VRC01-class precursors to GCs, where they undergo SHM, including VRC01-class mutations (26), which forecasted the successful VRC01-class priming by eOD-GT8 60mer/AS01B in the IAVI G001 clinical trial (11). The three mouse models used in that study, CLK19 (N6 subclass), CLK09 (N6 subclass), and CLK21 (PCIN63 subclass), bear genuine human BCRs identified from healthy donors (29). To investigate whether mRNA-LNP delivery of eOD-GT8 60mer would also effectively prime B cell responses in these models, we transferred naïve B cells bearing human CLK19 BCRs (eOD-GT8 K_D_: 1.8 μM) to recipient mice to produce precursor frequencies at either a ratio used in prior studies (26) or a more stringent precursor frequency in line with human physiology (29, 51, 52). We then compared immunization with eOD-GT8 60mer mRNA-LNP at a high (10 μg) or low (0.6 μg) dosage to eOD-GT8 60mer protein (Fig. 1A). All regimens induced similar GC formation in lymph nodes and spleens at day 14 (Fig. 1B and fig. S1, A and B). Low doses of eOD-GT8 60mer mRNA-LNP induced comparable antigen-specific CD45.2 responses to the eOD-GT8 60mer protein, no matter the starting precursor frequency (Fig. 1, B and C). At stringent, physiological precursor levels, the high dose of mRNA-LNP recruited more CD45.2 B cells to splenic GCs than either the low dose of mRNA-LNP or the eOD-GT8 60mer protein, though responses were indistinguishable in mice with a higher starting precursor frequency (Fig. 1C). No CD45.2 response was induced in eOD-GT8-knockout (KO) 60mer groups (Fig. 1, B and C). Thus, eOD-GT8 60mer mRNA-LNP and protein both recruited CLK19 B cells to GCs, but a high dose of mRNA-LNP improved responses at lower starting frequencies.

To determine whether we could develop a substantial long-term CD45.2 GC population as a precursor to boosting, we investigated GC kinetics in response to the high mRNA-LNP dose over time. In both lymph node and spleen, mRNA-LNP not only primed but also sustained GC responses until day 42 (fig. S1, C and D). At physiological frequency, compared to eOD-GT8 protein, mRNA-LNP recruited a significantly higher percentage of CD45.2 cells to GCs in spleens, though there was no difference at the higher frequency or in lymph nodes (Fig. 1D).

MBC formation is of particular importance to vaccine effectiveness (53). We found that mRNA-LNP induced a significantly higher percentage of class-switched IgG MBCs (CSM) than eOD-GT8 60mer protein at either starting precursor frequency (Fig. 1E and fig. S1, E and F). The antibody titers were also monitored until day 72; in the higher frequency cohort, mRNA-LNP elicited higher titers than protein at all time points. At physiological frequency, the immunogens were indistinguishable until day 72, at which point mRNA-LNP elicited significantly higher titers (Fig. 1F). Thus, mRNA-LNP induces and maintains B cell responses with similar or higher efficiency than protein in the CLK19 model—particularly at more stringent frequencies—and drives greater MBC formation.

### eOD-GT8 60mer mRNA-LNP recruits and maintains diverse VRC01-class precursors in GCs

VRC01-class precursors in the human repertoire belong to the same VH1–2 family, with precursor affinity varying on the basis of factors including Vκ or V_λ_ family (29, 51, 52). We used two additional CLK knock-in mouse models—CLK09 (Vκ1–33, eOD-GT8 K_D_: 350 nM) and CLK21 (Vκ1–5, eOD-GT8 K_D_: 440 nM)—alongside the aforementioned CLK19 model (Vκ1–33, eOD-GT8 K_D_: 1.8 μM) to test eOD-GT8 60mer mRNA-LNP priming; all three can be primed efficiently using the eOD-GT8 60mer protein (26).

**Fig. 2.**
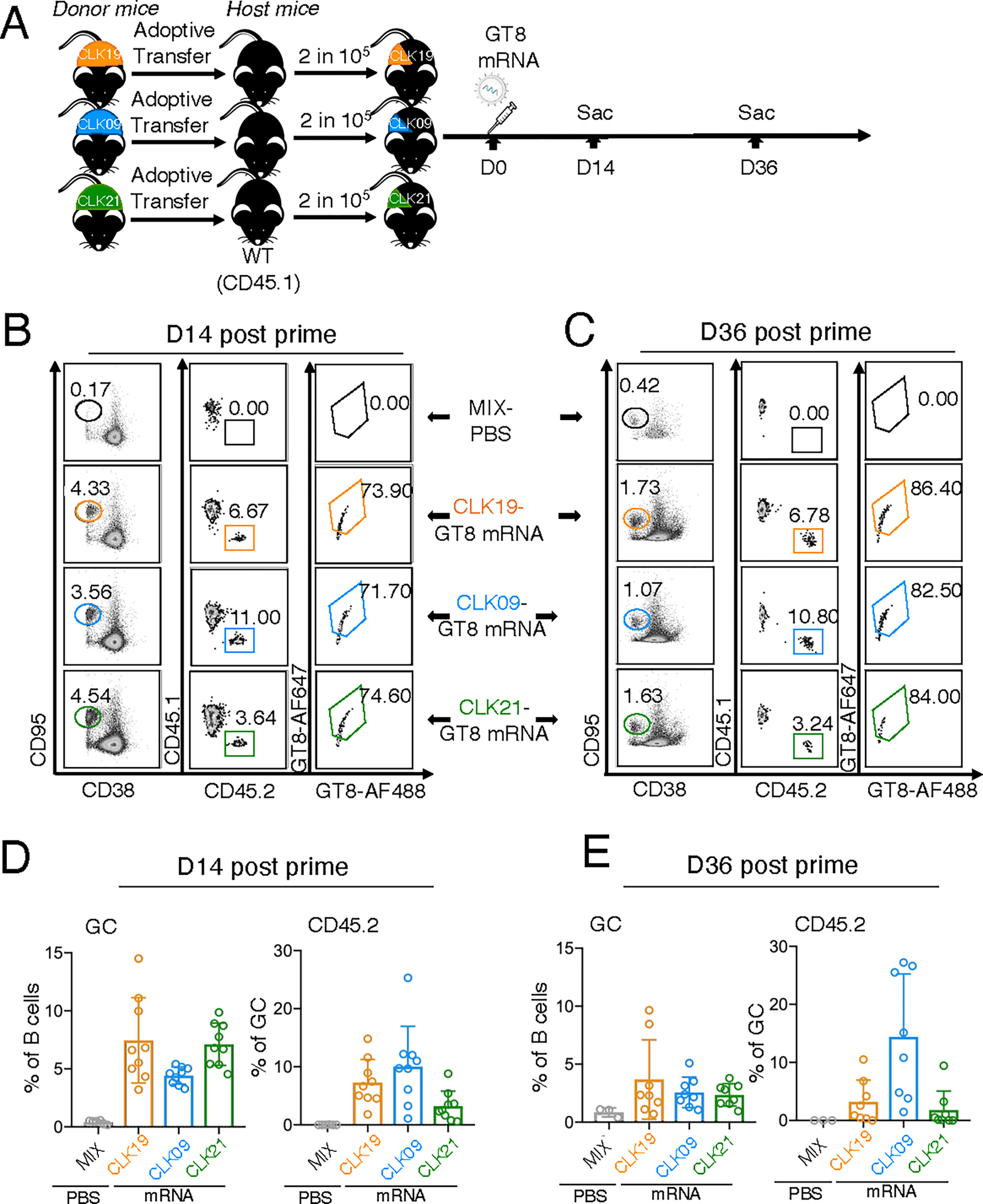
GT8 60mer mRNA-LNP recruits and maintains diverse VRC01-class precursors in GCs. (**A**) Schematic of adoptive transfer and immunization for CLK19, CLK09, and CLK21. Eight-week-old WT CD45.1 mice were singly adoptively transferred with B cells from CLK19/CLK09/CLK21 KI CD45.2 mice to establish precursor frequencies of ~2 in 10^5^ B cells, then immunized with 10 μg of eOD-GT8 60mer mRNA-LNP (Day 0). Mice adoptively transferred with all CLKs were immunized with PBS as control. LN were isolated for analysis at days 14 and 36. (**B** and **C**) Frequency of CD45.2 antigen specific (Ag^+^) binders within GCs in individual CLK adoptively transferred mice (ATM) at days 14 (B) and 36 (C). Gated plots represent the percentage of GC (first column), and CD45.2 in GCs (second column) and the antigen specific fraction of that group (third column), respectively. (**D** and **E**) Quantification of the frequency of CD45.2 within GC in individual ATM. The graphs show the quantification of GC in B cells (left) and the CD45.2 frequency within GC (right) from lymph nodes at day 14 (D) and 36 (E). The *x*-axis indicates the cohort and the *y*-axis represents the percentage of GC out of B cells (left) or CD45.2 B cells within GC (right). Two independently repeated experiments were analyzed. n=3–5 mice per independent group. Bars indicate geometric means and geometric SD of mice from pooled groups.

Mice were adoptively transferred with CLK19, CLK09 or CLK21 precursors and immunized as shown (Fig. 2A). At day 14, mRNA-LNP induced GC formation (means: CLK19: 6.7%; CLK09: 4.4%; and CLK21: 7.1%) and CD45.2 B cell entry into GCs (means: CLK19: 7.3%; CLK09: 10.0%; and CLK21: 2.3%) in all models, whereas controls injected with PBS formed limited GCs containing no CD45.2^+^ cells (Fig. 2, B and D). Additionally, although GC size decreased on day 36 in all models (to approximately 2 to 4%), specific CD45.2 B cell responses in GCs were sustained until day 36 (Fig. 2, C and E). Thus, eOD-GT8 60mer mRNA-LNP can prime and sustain distinct precursor lineages with variable affinities.

### Initial affinity and precursor frequency are not the sole determinants of GC occupancy

Previous work on eOD-GT immunogens found that VRC01-class precursors with low affinity (GT1: 40 μM; GT2: 14 μM) can be primed at high precursor frequency (1 in 10^3^), whereas at lower precursor frequencies (1 in 10^5^ to 1 in 10^6^), only high-affinity precursors (GT5: 0.5 μM) can be primed robustly. This suggests that competitive fitness within the GC is dependent on both affinity and precursor frequency, even with the high avidity provided by 60mer nanoparticles (37). In humans, VRC01-class precursors are rare, at approximately 1 in 3×10^5^ (29, 51, 52). Therefore, affinity and frequency-based competition between cognate B cells within the GCs may be strong determinants of the B cell population activated by priming.

**Fig. 3.**
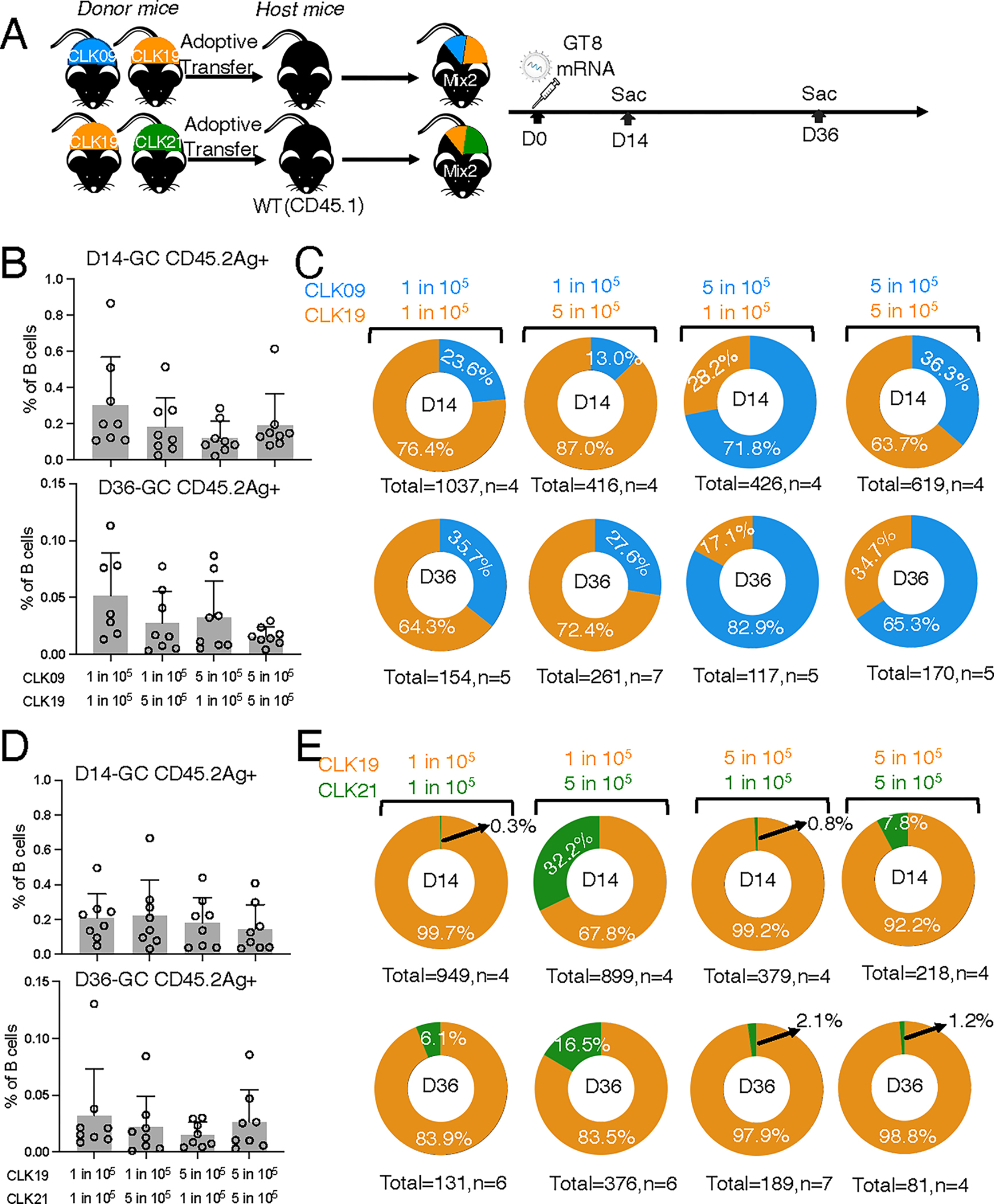
GC responses induced by mRNA-LNP in mice bearing two types of CLK BCRs. (**A**) eOD-GT8 mRNA-LNP immunization of 8-week-old CD45.1 mice adoptively transferred with two CLK B cell lines at variable ratios. (**B**) Quantification of GC CD45.2 binders out of total B cells in CLK09–19 adoptive transfer recipients at days 14 (upper) and 36 (lower). The *x*-axis indicates the immunized group with varying CLK09/CLK19 precursor ratio and *y*-axis represents the percentage of GC CD45.2 binders out of B cells. Two independently repeated experiments were analyzed. n=3–4 mice per independent group. Bars indicate geometric means and geometric SD of mice from pooled groups. (**C**) Sequence frequency of CLK lineages at day 14 (top) and 36 (bottom). Antigen-specific CD45.2 were bulk-sorted for 10x sequencing analysis. Pie charts represent the frequency of CLK09 (blue) and CLK19 (orange) lineages from each immunized group with varying precursor ratios. Total: sequences per group; n: mice per group. (**D**) As in (B), but for CLK19–21. (**E**) As in (C), but for CLK19–21. (B and C) (i) both lines at 1 in 10^5^ (both low); (ii) CLK09 at 1 in 10^5^ and CLK19 at 5 in 10^5^ (CLK19 high); (iii) CLK09 at 5 in 10^5^ and CLK19 at 1 in 10^5^ (CLK09 high); and (iv) both lines at 5 in 10^5^ (both high). (D and E) identical ratios were established for CLK19 and CLK21. LNs were analyzed at days 14 and 36 after immunization.

To investigate post-prime competition in GCs, we used adoptive transfer to generate recipient mice with CLK09 plus CLK19 (CLK09–19) or CLK19 plus CLK21 (CLK19–21) at four different ratios (Fig. 3A). In all groups, comparable numbers of total antigen-specific CD45.2 B cells were found in GCs at days 14 and 36 (Fig. 3, B and D). However, when GC antigen (Ag)^+^ CD45.2 B cells were sorted for 10x single-cell sequencing (fig. S2A), CLK19 in CLK09–19 mice comprised the larger fraction of all sequences in most treatments and time points. The cohort with CLK09 initially high and CLK19 low was the only exception, with CLK09 cells predominant (CLK09: 71.8% at 14 days and 82.9% at 36 days). When both CLK09 and CLK19 were at the higher starting precursor frequency, CLK19 was initially more frequent at 14 days (CLK19: 63.7%) but not at 36 days (CLK19: 34.7%) (Fig. 3C). However, significant differences per mouse were due to higher starting frequencies, rather than lineage (fig. S2B).

By contrast, CLK19 comprised most of GC B cells at all starting frequencies and time points in the CLK19–21 treatments (Fig. 3, D and E, and fig. S2C). Intrinsic features apart from the reasonable affinity of CLK21 may explain its relatively poor sustenance; competition would be unlikely to be the only cause, as CLK21 exhibited similarly low rates of GC entry after priming in singly adoptively transferred mice (Fig. 2, D and E).

Thus, for precursors within a narrow affinity band (0.35–2 μM), GC prevalence after priming seems to be driven more by initial frequency (CLK09 and CLK19) or intrinsic qualities (CLK21) other than epitope affinity. Furthermore, the lack of an exclusionary competitive effect demonstrates the feasibility of activating multiple precursor lineages in the same host.

### Diverse CLK lineages in a mini-repertoire undergo SHM and affinity maturation after eOD-GT8 60mer mRNA-LNP priming

**Fig. 4.**
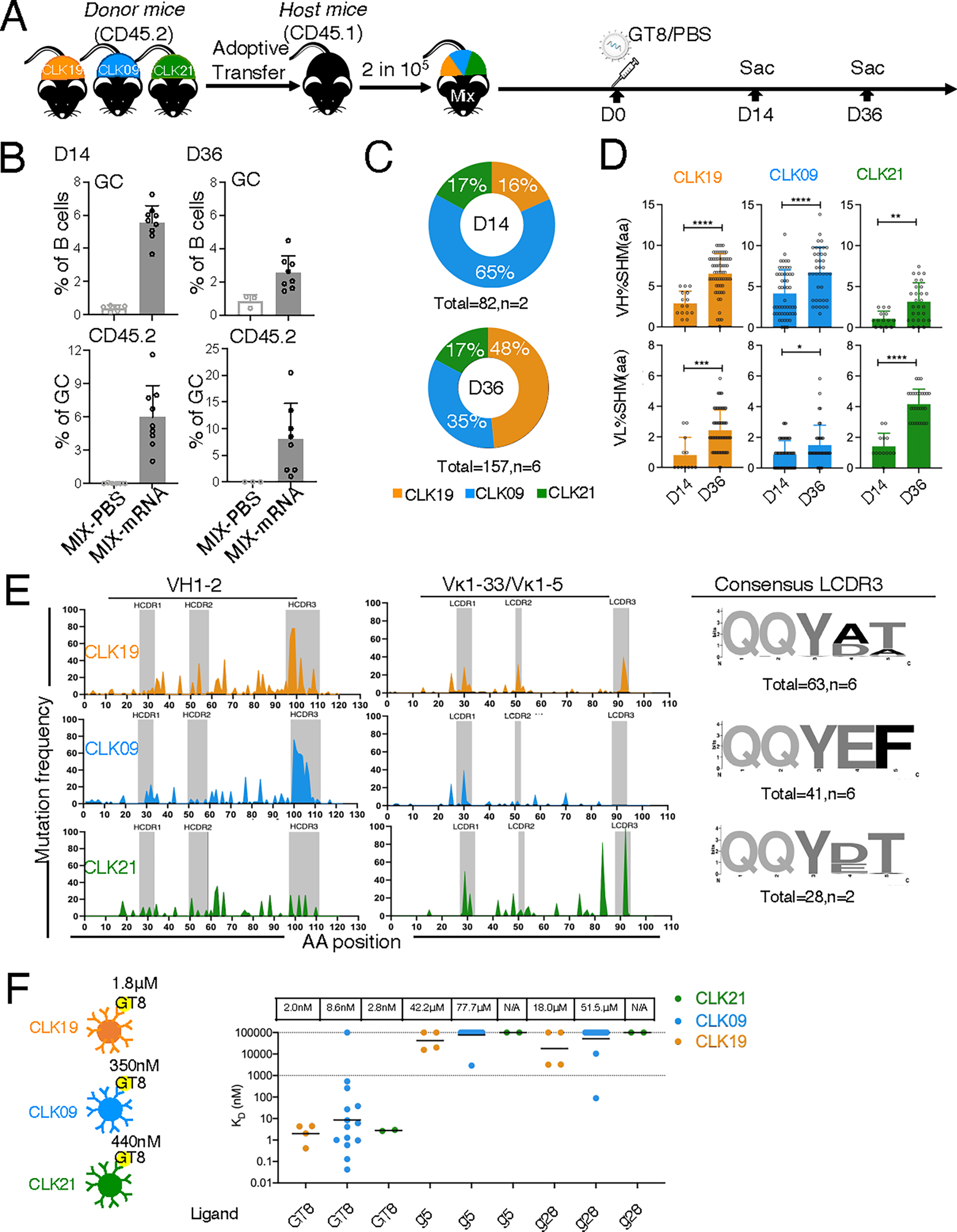
GC responses induced by eOD-GT8 60mer mRNA-LNP in CLK mice bearing human mini-B cell repertoire. (**A**) Schematic: 8-week-old WT CD45.1 mice received B cells from CLK19, CLK09, and CLK21 KI CD45.2 mice in tandem to establish a mini-human repertoire with precursor frequencies of 2 in 10^5^ B cells (approximately 6 in 10^6^ each), then immunized with 10 μg of eOD-GT8 60mer mRNA-LNP one day post transfer. Mice adoptively transferred with all CLKs were immunized with PBS as control. LN were isolated on days 14 and 36 after immunization. (**B**) Quantification of GC B cells (upper) and GC CD45.2 B cells (lower) on days 14 (left) and 36 (right). The *x*-axis represents the immunized group and the *y*-axis represents the percentage. Two independent experiments were analyzed. n=3–5 mice per independent group. Bars indicate geometric means and geometric SD from mice in pooled groups. (**C**) Single cell–plate sequencing was performed for CD45.2 Ag^+^ binders from GCs at day 14 (upper) and 36 (lower). CLK09 (blue), CLK19 (orange), and CLK21 (green) sequences from paired heavy chain (HC) and light chain (LC) were used for sequence frequency analysis. Total: sequences per group; n: mice per group. (**D**) Percent amino acid (aa) change in IGHV (upper) and IGLV (lower) of CLK19, CLK09, and CLK21 lineages isolated from GC at days 14 and 36. Data from two independent experiments were analyzed. Each dot represents one sequence. Bars indicate geometric means and geometric SD of 12–63 sequences per group from pooled experiments. Significance was calculated with Student’s *t* test, *P<0.05, **P<0.01, ***P<0.001, and ****P<0.0001. (**E**) Mutation frequency was assessed via hotspot analysis for both heavy (left) and light (right) antibody chains; WebLogo (88) at right. (**F**) SPR affinity of post-prime mAbs for GT8-prime and g5- and g28-boost ligands. Top values are the geometric means of mAb affinity after priming. Left values are the geometric means of affinity for CLK GL mAbs to eOD-GT8 ligand. Each dot represents one mAb, 2–14 mAbs per group were analyzed from a single SPR experiment.

As there are precursors of varying affinities targeting eOD-GT8 60mer in the human repertoire (11, 29, 51, 52), whether eOD-GT8 60mer mRNA-LNP can induce responses for diverse CLK precursors in a complex competitive system within the same host—as the nanoparticle did in the G001 clinical trial (11)—could be a key determinant of clinical efficacy. We simultaneously adoptively transferred CLK19, CLK09, and CLK21 CD45.2 precursors into recipients to generate a mouse bearing a mini-human B cell repertoire with all three CLK precursors at a precursor range in line with human physiological frequency (29), then primed with eOD-GT8 60mer mRNA-LNP (Fig. 4A). In the mRNA-LNP vaccination group at day 14, robust GCs formed (6% of B cells); there was almost no response detected in the PBS control group (figs. S3A and 4B). Although GC size after mRNA-LNP vaccination decreased to 2% at day 36, CD45.2 cells were approximately 6% of GC B cells at both timepoints (Fig. 4B and fig. S3A). Using single-cell sorting of antigen-specific CD45.2 B cells, we found that all three CLK lineages were induced by eOD-GT8 60mer mRNA-LNP immunization at day 14 (CLK19: 16%; CLK09: 65%; and CLK21: 17%) and sustained until day 36 (CLK19: 48%; CLK09: 35%; and CLK21: 17%) (Fig. 4C).

Antigen-driven affinity maturation of the BCR results from the accumulation of SHM via activation-induced cytidine deaminase (AID) and cellular selection in GCs (54). We used single-cell sequencing to assess SHM accumulation and affinity maturation in these CLK lineages over time after mRNA-LNP priming. All lineages underwent substantial diversification (fig. S3B). Both heavy chain (HC) and light chain (LC) exhibited SHM in all CLK lineages at day 14 with mean HC mutation frequencies of 2.9% aa/1.3% nt (CLK19), 4.2% aa/3.7% nt (CLK09), and 1.1% aa/0.6% nt (CLK21) and LC frequencies of 0.8% aa/0.4% nt (CLK19), 0.9% aa/0.5% nt (CLK09), and 1.4% aa/0.8% nt (CLK21) (Fig. 4D, fig. S3C and table S1). The mutation frequency in both HC and LC significantly increased two-to-three fold at day 36 (CLK19: 6.5% aa/3.0% nt, CLK09: 6.7% aa/4.7% nt and CLK21: 3.2% aa/1.8% nt for HC; CLK19: 2.4% aa/1.8% nt, CLK09: 1.5% aa/0.9% nt and CLK21: 4.2% aa/2.1% nt for LC), indicating that SHM accumulates in both CLK HCs and LCs over time (Fig. 4D, fig. S3C and table S1).

To further understand SHM distribution, we analyzed the mutation frequency in each aa position at day 36, finding it higher in the CLK19 and CLK09 heavy chain complementarity determining region (HCDR) 3s. Indeed, some sites reached mutation rates of nearly 80%, whereas the highest CLK21 peak was approximately 20% (Fig. 4E). All CLK LCs exhibited SHMs in light chain complementarity determining region (LCDR) 1 (Fig. 4E). Mutations and deletions in this region are key to avoiding steric clash with N276 and N462 glycans in the mature bnAb (14). In the LCDR3, most VRC01-class B cells exhibit a 5-aa motif (CQQYXXF), which is important for Env contact (11, 15, 55). In CLK19, we observed 40% of As at motif position 4 switch to D and 23% of Ts at motif position 5 switch to A. For CLK21, 100% of Gs at motif position 4 switch to D or E, though there is no mutation in motif position 5 (Fig. 4E). No mutation occurred in the LCDR3 of CLK09, where the 5-aa motif is identical to mature VRC01. To test whether accumulated SHM facilitate affinity maturation, we expressed mAbs from days 14 and 36 and performed SPR to the eOD-GT8 ligand, the mean of each CLK lineage underwent 40–900-fold increases in affinity after priming (Fig. 4F).

To move our model to the next GT vaccination stage, we designed a series of booster candidates based on a stabilized native HxB2 gp120 core lacking the N276 glycan (HxB2–2CC-core-N276D) (56) and then measured their affinity to post-prime mAbs. Post-prime, a few CLK19 and CLK09 lineages exhibited weak affinity to the g5 (CLK19: mean of 42.2 μM and CLK09: mean of 77.7 μM) and g28 (CLK19: mean of 18.0 μM and CLK09: mean of 51.5 μM) boosters (Fig. 4F), suggesting that post-prime affinity maturation may allow these mAbs to target boost immunogens.

Thus, eOD-GT8 60mer mRNA-LNP primes diverse CLK precursors with variable affinities. Furthermore, the post-prime mAbs not only acquire 40–900-fold increases in affinity to eOD-GT8 ligand, but also exhibit weak affinity to booster ligands.

### Prime–boost strategy drives the evolution of CLK precursors toward mature-like VRC01 bnAbs

**Fig. 5.**
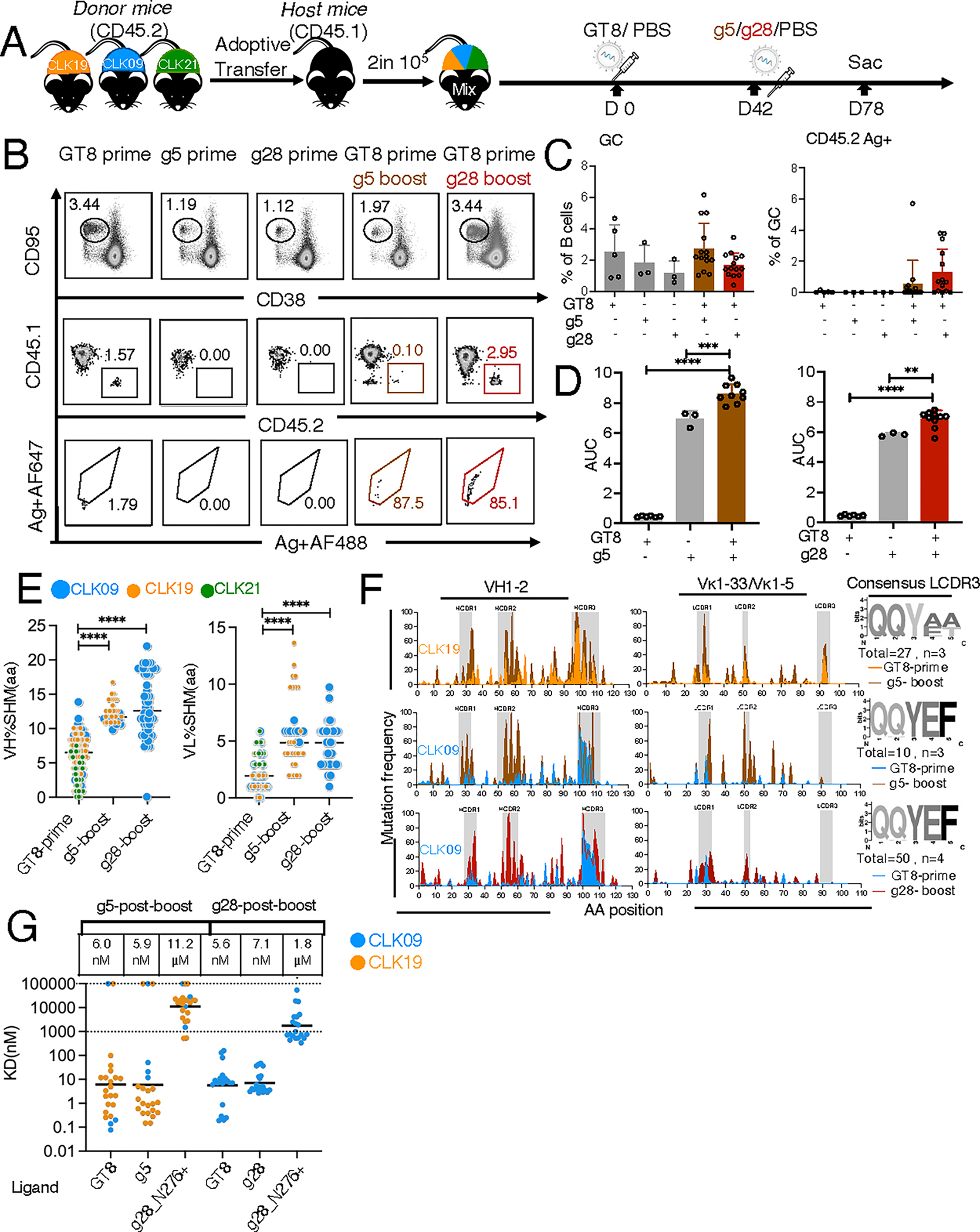
GC responses and sequence evolution triggered by g5 and g28 boosters. (**A**) Schematic: mini-repertoire was established as in Fig. 4A. mRNA-LNP doses were 10 μg for prime and boost. (**B**) Representative flow cytometry plots of antigen-specific GC CD45.2 responses after prime–boost strategy. A probe including both g5 and g28 was used to detect CD45.2 binders from the GT8-prime group. Probes of g5 or g28 alone were used to detect binders after g5-prime–boost or g28-prime–boost, respectively. Ag^+^CD45.2 binders were gated on SSL^+^/B220^+^/CD95^+^CD38^−^/CD45.2^+^/Ag^+^. GC percentage (upper row), CD45.2 (middle row), and Ag^+^CD45.2 (bottom row). (**C**) Quantification of GC B cells (left) and GC Ag^+^CD45.2 (right) at D78. The *x*-axis represents the immunized group and the *y*-axis represents the percentage of GC B cells (left) and Ag^+^CD45.2 within GCs (right). Circles represent one mouse. Two independently repeated experiments were pooled for analysis. n=3–7 mice per independent group. (**D**) ELISA-derived specific IgG titers post GT8/g5/g28-prime and post-g5 and g28 boost. Plates were coated with g5 (left) or g28 (right) ligands. Two independent experiments were pooled for analysis. n=3–5 mice per independent group. The *x*-axis represents the immunization groups and the *y*-axis AUC. (**E**) Percent aa SHM in IGHV (left) and IGLV (right) of CLK19 (orange), CLK09 (blue), and CLK21 (green) lineages from GT8-prime (36 days post prime; reproduced from Fig. 4C day 36 for comparison) and g5-or g28-boost (36 days post boost) groups. Each dot represents one sequence, 37–130 sequences per group from two independent experiments were pooled for analysis. (**F**) Mutations were assessed via hotspot analysis for HC (left) and LC (right). CLK19 (orange) and CLK09 (blue) lineages from GT8-prime only group (prime-only group reproduced from Fig. 4C day 36 for comparison); g5-boost (brown) and g28-boost (red). WebLogos at right (88). (**G**) Affinity of mAbs after boosting to GT8, g5 g28, or g28_N276^+^. Top values are the geometric means of mAb affinity. Each dot represents one mAb, 21–23 mAbs per group were analyzed from a single SPR experiment. Where used, bars indicate geometric means and geometric SD of pooled mice and significance was calculated with one-way ANOVA: ** *P*<0.01, ***P<0.001, and ****P<0.0001.

Having demonstrated that some CLK clones gained affinity to boost candidates after priming, we next investigated whether these boosters could extend or reactivate the GC response and further affinity maturation. We primed the mini-CLK B cell repertoire with eOD-GT8 60mer mRNA-LNP as above, boosted with g5 60mer or g28 60mer mRNA-LNP at day 42, and analyzed GCs at day 78 (Fig. 5A). All immunization regimens—including prime-only groups using either eOD-GT8 or one of the boost candidates (g5 and g28), as well as the prime–boost sequences of eOD-GT8 followed by either a g5 or g28 boost—led to comparable GC formation (Fig. 5B and Fig. 5C). However, antigen-specific CD45.2 responses were induced in more mice after the eOD-GT8/g5 (with responses in five out of 14 mice) and eOD-GT8/g28 (nine of 14 mice) heterologous prime–boosts relative to the prime-only approach (Fig. 5B and Fig. 5C). A comparative boosting experiment was performed for mice singly transferred with CLK19, CLK09, or CLK21 (fig. S4A), and antigen-specific responses were elicited in the cohorts adoptively transferred with CLK19 or CLK09 (fig. S4, B and C). However, among CLK21 recipients, only one mouse generated an antigen-specific response after g28 boosting and there was no significant response to g5 (fig. S4, B and C). Furthermore, heterologous prime–boost elicited significantly higher antibody titers than the prime-only groups (Fig. 5D). Thus, both boost immunogens may enhance some, but not all, CLK responses.

We next tested whether boosting furthered bnAb-like maturation. SHM frequency in VH and VL was much higher in the CLK19 and CLK09 sequences recovered 36 days after administration of the g5 boost compared with 36 days after the eOD-GT8 prime alone. After the g28 boost, almost all recovered sequences were CLK09, which exhibited significantly more SHM than the eOD-GT8-prime group (Fig. 5E and fig. S5, A and B), suggesting differential SHM accumulation based on both lineage and boost immunogen. In the key HCDRs, both CLK19 and CLK09 exhibited much higher mutation frequency post-g5 boost than after eOD-GT8-prime alone (Fig. 5F), particularly in HCDR2, which is important for Env contact (14). VRC01 HC mutations occurred post-boost in both CLK19 (after g5) and CLK09 (after g5 or g28) in key residues, including K19R, S54Y, T57V, Q61R, G65D, S83R, K85R, and K100bW. Furthermore, in CLK19, ~46% of Es occurring at the aa position 4 of the 5-aa motif in LCDR3 described above were identical to the mature VRC01 (Fig. 5F). After priming alone, this mature mutation was never observed (Fig. 4E). As the QQYEF motif matches mature VRC01 in CLK09, this lineage did not accumulate similar mutations. Moreover, phylogenetic trees for g5 and g28 vaccinated groups demonstrated substantial evolution after boosting beyond that provided by priming alone (fig. S5C). Thus, g5 and g28 trigger further evolution, including the acquisition of mature-like SHM, in some CLK lineages.

Previously, we found that some post-prime mAbs possessed weak affinity to the g5 or g28 ligand (Fig. 4F). To determine whether mAbs after boosting undergo affinity maturation to the boost, we tested SPR affinity at day 78. mAbs from the CLK09 and CLK19 lineages in the g5-boost-group not only acquired high affinity to eOD-GT8 (mean of CLK19 and CLK09: 6.0 nM), but also a 7000–13,000-fold increase in affinity to g5 (mean of CLK19 and CLK09: 5.9 nM) relative to the eOD-GT8-prime-only group (CLK19: 42.2 μM; CLK09: 77.7 μM) (Figs. 4F and 5G). In the g28-boost-group, CLK09-lineage mAbs similarly achieved 5.6 nM affinity to eOD-GT8 and 7.1 nM affinity to g28 (Fig. 5G), though the mean of affinity from the eOD-GT8-prime-group to g28 only reached 51.5 μM (Fig. 4F). The mAbs from both g5 and g28-boost cohorts also achieved affinity (11.2 μM and 1.8 μM) to g28_N276+, a variant of g28 that includes the important N276 glycan in the CD4bs (Fig. 5G). Thus, these mRNA-LNP booster candidates could trigger affinity maturation toward mature-like VRC01 bnAbs in a competitive GC environment, which affirms that mice bearing human-mini-B cell repertoires may serve as preclinical models for boost-stage HIV immunogens.

### The improved booster g28v2 drives a VRC01-like response

**Fig. 6.**
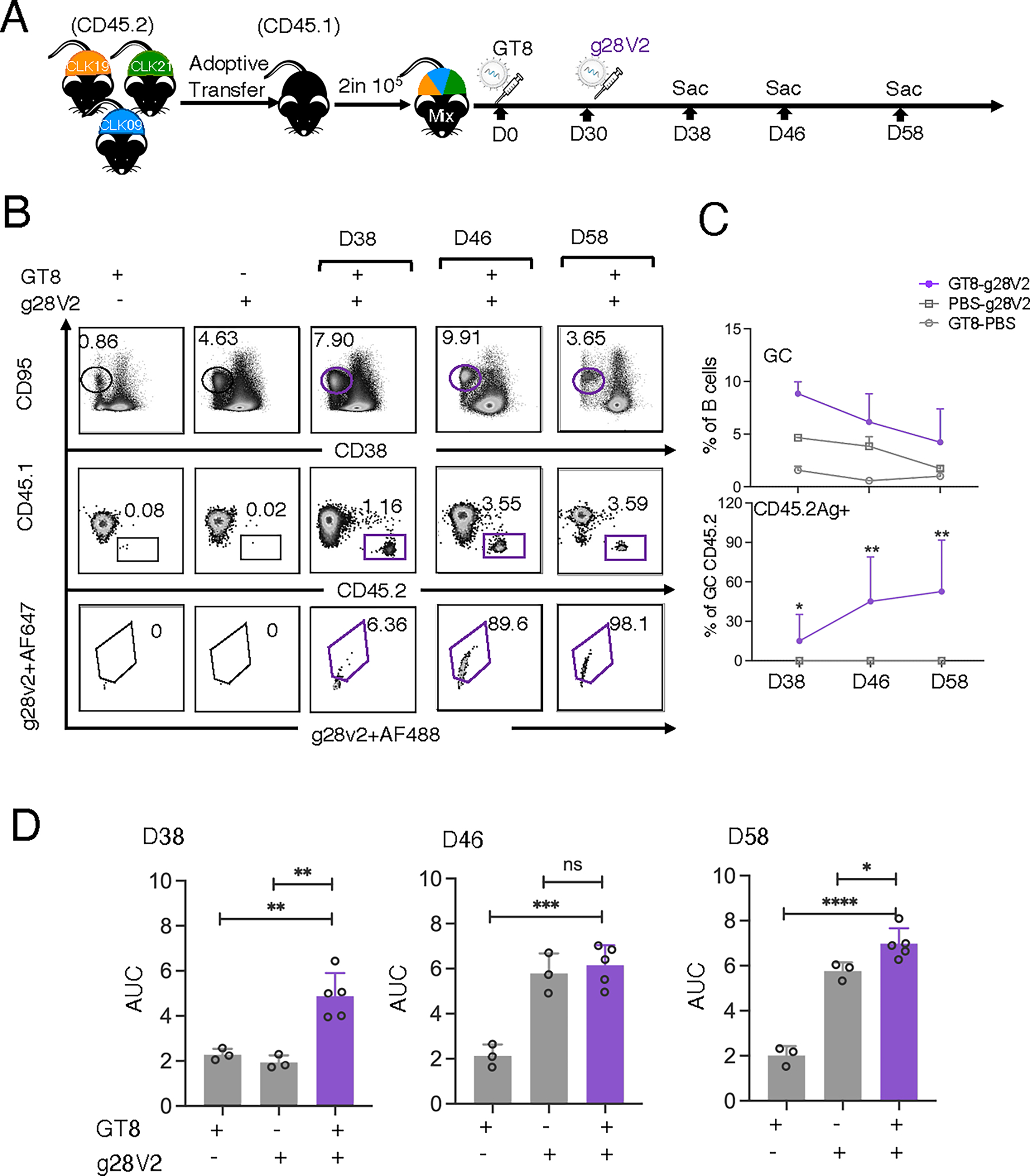
GC responses after g28v2 boosting. (**A**) Schematic of mini-repertoire adoptive transfer to evaluate GT8 prime (Day 0) followed by g28v2 boost (Day 30). The mini-repertoire was established as in Fig. 4A, and the dosages of mRNA-LNP for both prime and boost are 10 μg. (**B**) Flow cytometry of CD45.2 binders within LN GCs over time after g28v2 boosting. g28v2 probe was used to detect CD45.2 binders. CD45.2 binder populations were gated on SSL^+^/B220^+^/CD95^+^CD38^−^/CD45.2^+^/Ag^+^. Gated plots represent the percentage of GC (upper row), CD45.2 (middle row) and g28v2 specific CD45.2 (lower row). (**C**) Quantification of GC B cells (upper) and GC CD45.2 binders (lower) over time after g28v2 boosting. The *x*-axis represents the time points for boosting and the *y*-axis represents the percentage of GC B cells (upper) and GC CD45.2 binders (lower). Two independently repeated experiments with 3–5 mice per independent group were analyzed. Each circle or square with bars indicates geometric means and geometric SD from mice in pooled groups. (**D**) ELISA-derived specific IgG titers post GT8/g28v2-prime and g28v2 boost over time; Plates were coated with g28v2 ligands for ELISA detection. n=3–5 mice in a single experiment. Bars indicate geometric means and geometric SD. The *x*-axis represents the immunization groups and the *y*-axis represents the AUC. Significance was calculated with one-way ANOVA and shown as: ns *P*>0.05, *P<0.05, **P<0.01, ***P<0.001, and ****P<0.0001.

We used g28 as the basis for the design of another mRNA-LNP booster, g28v2 60mer, now in the IAVI G002 clinical trial (NCT05001373). This immunogen contains an N-linked glycosylation site shift from HxB2 position 399 to 397 and a modified linker between the lumazine synthase nanoparticle and the gp120 core domain relative to g28 60mer, increasing expression and particle formation (56). To evaluate g28v2 60mer in preclinical mouse models, we performed mini-repertoire experiments as above (Fig. 6A). After boosting, as the frequency of total GC B cells decreased over time, the frequency of antigen-specific binders conversely increased in those GCs (Fig. 6, B and C). There were no binders in eOD-GT8 or g28v2 prime-only control groups post boost (Fig. 6C). g28v2 somewhat improved CD45.2 maintenance compared with g28 (fig. S6A–B). Relative to prime-only cohorts, specific antibody titers after a g28v2 60mer mRNA-LNP boost were significantly improved (Fig. 6D). Thus, the g28v2 boost is highly effective at specific activation of lineages primed by eOD-GT8.

**Fig. 7.**
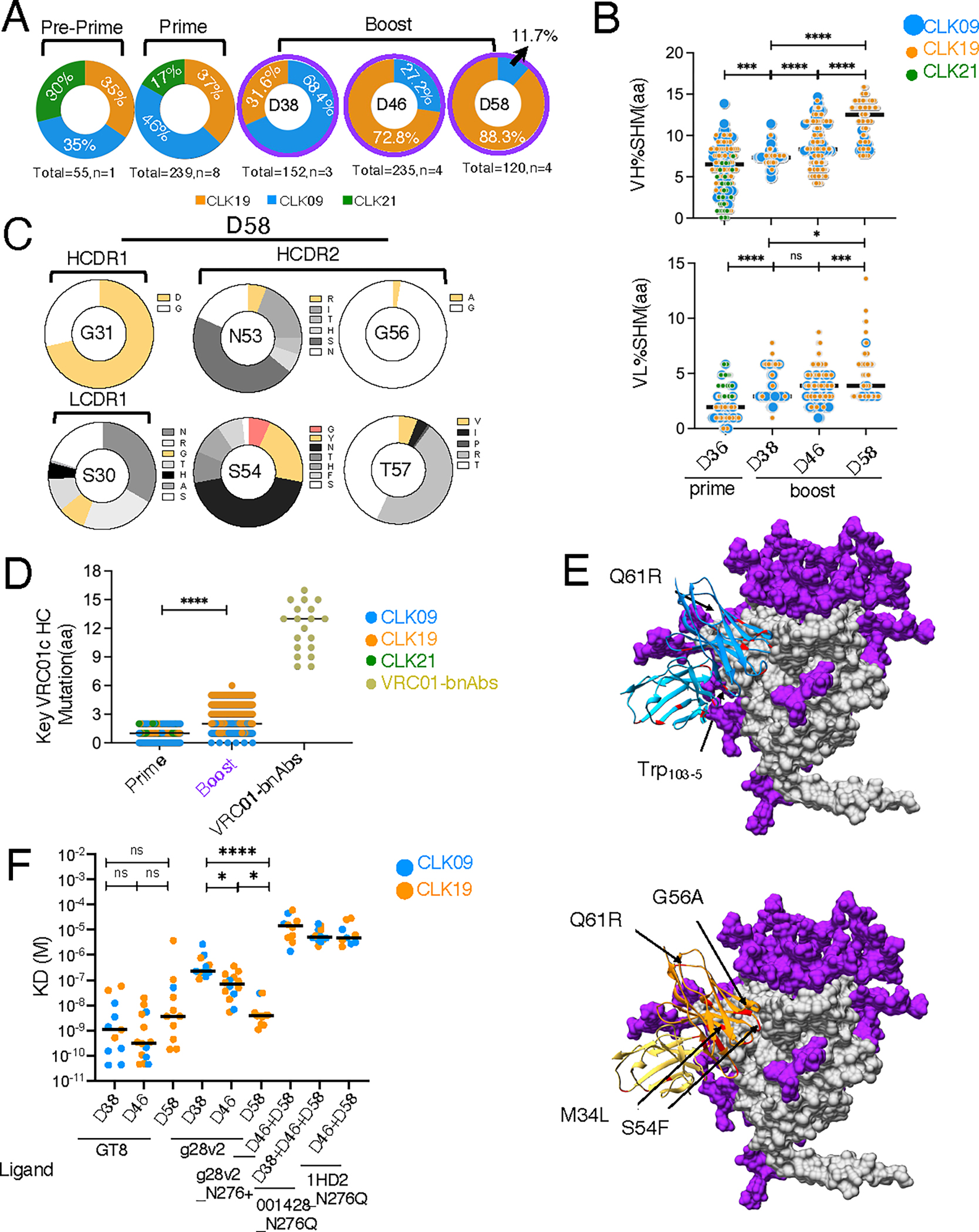
SHM and affinity maturation after g28v2 boosting. (**A**) Sequence frequency of CLK lineages in GC. CLK09 (blue), CLK19 (orange), and CLK21 (green). Single-cell-plate sequencing was used as in Fig. 4C. Total: sequences per group; n: mice per group; prime data combines sequences from days 14 and 36 shown in Fig. 4C. (**B**) Percent aa SHM in IGHV (upper) and IGLV (lower) of CLK19 (orange), CLK09 (blue) and CLK21 (green) lineages from GT8-prime- (day 36 data reproduced from Fig. 4C) and g28v2-boost-groups. Each dot represents one sequence, and 118–240 sequences per group from two independent experiments were pooled for analysis. (**C**) Mutation frequency of key residues in CDRs of HC and LC after g28v2 boosting. Key mutations on D58 were analyzed referring to the mature VRC01-class bnAbs. Yellow indicates the aa is identical to bnAb VRC01 (14), whereas red signifies identity to bnAb N6 (27). (**D**) Key mutation frequency in post-boost HCs, in comparison to mature VRC01-class bnAbs. Each dot represents one mAb. The *x*-axis represents immunization group or a representative panel of 19 VRC01-class bnAbs (12A12, 12A21, N6, VRC27, N17, N60P1.1, N60P25.1, N60P23, PCIN63_71l, PCIN63_66B, PCIN63_71G, NIH45–46, VRC07b, VRC23, VRC01, VRC02, VRC18, VRC08, VRC-PG19) with minimal (≤ 3 aa) indels and the *y*-axis quantifies aa key mutations in HC. (**E**) Computational molecular modeling of mAb Fvs from CLK09 lineage at D58 (upper) and CLK19 lineage at D46 (lower) binding to g28v2 was performed by AlphaFold2 and Rosetta. Purple represents N-linked glycans, gray g28v2 peptide, dark blue and light blue (upper) represent the HC and LC of CLK09 Fv regions, respectively, orange and yellow (lower) represent the HC and LC of CLK19 Fv regions, respectively, and red represents the mutations from germline. Arrows denote key VRC01-class HC mutation. (**F**) Affinity of mAbs to ligands (GT8, g28v2, g28v2_N276+, 001428_N276Q trimer and 1HD2_N276Q trimer) after boosting over time. Black lines represent the geometric means of mAb affinity post-boost. Each dot represents one mAb, 9–14 mAbs per group from a single SPR experiment were analyzed. Significance was calculated with Student’s *t* test (D) or one-way-ANOVA (B and F) and shown as: ns *P*>0.05, *P<0.05, ***P<0.001, and ****P<0.0001.

To determine whether the VRC01 developmental path was maintained, we analyzed BCR modification and affinity gains. SHM was enhanced relative to prime-only in both the HC and LC of the CLK19 and CLK09 lineages, and it increased over time (Fig. 7, A and B, and fig. S7A and S7C). By contrast CLK21 was not an appreciable part of the post-boost population, which was consistent with the results of the g5 and g28 boosts (Fig. 5 and figs. S4 and S5). HCDR1, HCDR2 and LCDR1 mutation frequency increased relative to prime-only in both CLK19 and CLK09, as did LCDR3 in CLK19 (fig. S7B). At day 58, 5% of CLK19 LCDR3s mutated to the mature QQYEF motif (fig. S7B). Mutations also occurred at key residues in other regions: G31 in HCDR1 switched to D, which is a bnAb-type mutation (57), at all time points, with frequency increasing over time (Fig. 7C and fig. S8A and B). N53 and S54 mutations in HCDR2, which contribute to potency and breadth (7), accumulated over time. At day 46 and 58, N53 switched partly to R or Q, the mature aa in VRC01 (14, 57) or N6 (27), respectively, whereas at all time points, S54 switched partly to G (as in mature VRC01) and Y (as in mature N6) (Fig. 7C and fig. S8, A and B). G56A and T57V, associated with increased neutralization breadth against glycan-N276 bearing viruses (7, 58), were observed at days 46 and 58 (Fig. 7C and fig. S8, A and B). Mutations at these key residues accumulated over time (fig. S8C). S30G in LCDR1 was also observed after boosting, which may result in loop flexibility and is present in Vκ1–33 mature VRC01-class bnAbs (Fig. 7C and S8A–B).

Key VRC01-class mutations also occurred at additional HC positions, including 19, 34, 61, 81, and 82a (table S2). Across all heavy chain sites, g28v2 significantly increased the total number of key VRC01 class mutations compared to prime-only (Fig. 7D). Furthermore, some mAbs in the post-boost group almost achieved the minimal mutation level of a representative panel of 19 VRC01-class bnAbs with minimal (≤3 aa) indels (Fig. 7D). Thus, this prime–boost regimen represents an improvement over the first generation of boosters.

To visualize the mutations induced after g28v2 immunization and their potential interactions with g28v2, we performed computational molecular modeling of two antibody Fv regions, one from a CLK09 lineage and the other one from a CLK19 lineage, each bound to g28v2 (Fig. 7E). Key mutations present in both mAbs were at the binding interface with g28v2 (Fig. 7E).

To determine whether mAb SHM evolution facilitates affinity maturation, we tested the SPR affinity of mAbs to either the eOD-GT8-priming antigen, the g28v2-boosting antigen, or native-like Env trimer over time. mAbs from the CLK09 and CLK19 lineages in the g28v2-boost-group not only acquired high affinity to eOD-GT8 (D38: 1.1 nM, D46: 0.3 nM, D58: 3.7 nM), but also increased affinity to g28v2 over time (D38: 227 nM, D46: 67nM, D58: 3.9 nM) (Fig. 7F). Moreover, the mAbs from g28v2-boost cohorts also gained affinity to g28v2_N276+ (14 μM), which includes an important N276 glycan in the CD4bs (Fig. 7F). The mAbs also achieved affinities of 5 μM and 4.8 μM, respectively, to stabilized trimers based on isolates HIV-001428–2 (59) and 1HD2-preinf-051916-B4_S62 (60), in which asparagine was mutated to glutamine at the N276 glycan position (Fig. 7F) (56). Thus, the GT8-g28v2 prime–boost strategy successfully triggers mAb evolution and affinity maturation to more native-like immunogens.

## DISCUSSION

The COVID-19 epidemic moved the mRNA-LNP platform to the forefront of vaccine research, but specifically those vaccines expressing membrane-bound antigens (61, 62). In similar mouse models, HIV Env N332-supersite responses could be boosted via membrane-bound mRNA-LNP regimens initiated by a priming immunogen found effective in non-human primates (63, 64). Nanoparticle vaccines displaying a high density of antigens facilitate deposition within B cell follicles and induce a stronger immune response (65–68). We have now validated HIV GT prime–boost regimens of secreted soluble particles, including immunogens undergoing clinical trials (NCT05001373). A concurrent investigation of these regimens in a rearranging mouse model (69) was also encouraging (56). Furthermore, we found that mRNA-LNP priming induced an enhanced B cell immune response compared to protein, particularly in terms of MBC generation and antibody titers.

Our key preclinical findings are that with mRNA-LNP, multiple precursors to VRC01-class bnAbs can be (i) primed—consistent with clinical outcomes for the protein nanoparticle (11)—and (ii) boosted in the same animal. As humans have substantially more follicles per lymph node and precursors per individual compared to mice, these findings are encouraging for clinical feasibility. The substantial similarity between outcomes in the mini-repertoire and in recipients of only a single lineage matches some prior work on interclonal competition (70). Previous work has demonstrated that affinity and avidity for the antigen, as well as initial frequency, affect competitive fitness in the GC (37, 65). However, although GT8 60mer mRNA-LNP priming recruited diverse CLK precursors within a narrow affinity gap to the GC, GC occupancy by CLK21 cells was consistently lower than that of CLK19 post immunization irrespective of initial frequencies and even though the initial affinity of CLK21 to eOD-GT8 was 440 nM, approximately fourfold higher than that of CLK19 (1.8 μM). As this was recapitulated in single-transfer models, epitope competition is unlikely to be a driver. Within this narrow affinity gap, therefore, other intrinsic qualities of the B cells lines may overwhelm the effects of starting frequency and initial antigen affinity. One potential explanation is variable BCR surface density, which is known to affect activation (71), due to differential expression in the knock-in lines. Alternatively, the higher affinity of CLK21 may drive a greater proportion of CLK21 GC B cells to differentiate into plasma cells rather than occupying GCs (72–74). However, as the inferred germline affinities for MBCs and post-vaccination GC cells isolated in the G001 trial ranged quite high (11), rapid plasma cell differentiation may not be an assured fate for high affinity precursors.

Variability in GC occupancy after priming likely affects the boost phase; it is therefore unsurprising that, after immunization with three distinct booster candidates, only two lineages were responsive, with CLK21 functionally absent post-boost. One possible explanation is that the large CLK19 and CLK09 populations in GCs post-prime increased the likelihood that some would develop affinity for the boosters. Notably, both lineages acquired weak affinity for the boosters post-prime. This increased opportunity for affinity maturation may allow recalled CLK19 and CLK09 MBCs to form secondary GCs and undergo further affinity maturation (39). Alternatively, CLK21 lines may simply not generate the appropriate set of mutations to enter the post-boost reaction, irrespective of residency time.

One question regarding multi-stage sequential immunizations is whether limitations in MBC - to GCs (40, 75) might ratchet the diversity of participants ever downwards, causing a gradual decline of recalled responses. Although HIV GT immunogens reported in recent studies activated naïve B cell responses and generated VRC01-like SHMs in humanized mice (18, 19, 22, 24, 26, 76), VRC01-class bnAb-like evolution shepherded by boosting has been achieved in only a few studies and generally with more permissive mouse models (6, 7, 33). G56A and T57V in the HCDR2, which are associated with increased neutralization breadth against glycan-N276 bearing viruses (7, 58), have been previously observed in mice (6, 7), but have proven challenging to induce consistently. In this study, only one shot of booster led B cells to acquire affinity to the N276+ ligand and exhibit those two mutations. Furthermore, some LCDR3s of the CLK19 lineages mutated to the mature VRC01 motif, QQYEF (15, 55), after the g28v2 boost (this motif is already present in CLK09). These data imply, therefore, that mRNA-LNP regimens have the potential to guide the maturation of VRC01-class responses and render them susceptible to subsequent immunization with native-like trimers, providing the foundation for future sequential vaccine design.

## MATERIALS AND METHODS

### Mouse models

CLK21, CLK09 and CLK19 C57BL/6J (CD45.2) mice with B cells expressing humanized BCR were generated at the animal facility of the Gene Modification Facility (Harvard University) as previously described (26). Breeding for colony expansion and experimental procedures was performed in the Ragon Institute of Mass General, MIT, and Harvard. Male 7–12-week-old B6.SJL-*Ptprc*^*a*^ *Pepc*^*b*^/BoyJ mice (CD45.1) were purchased from Jackson Labs for the experiments. Mice were randomly assigned to groups and group size was selected based on prior work in the field with similar immunogens. No power analysis was performed. All experiments were approved by the Institutional Animal Care and Use Committee (IACUC) of Harvard University and Massachusetts General Hospital (MGH) and conducted in accordance with the regulations of the Association for Assessment and Accreditation of Laboratory Animal Care (AAALAC) under protocols 2016N000022 and 2016N000286.

### Adoptive transfer and immunization

CD45.2 B cells isolated from CLK21, CLK09, and CLK19 BCR KI mice using the Pan B Cell Isolation Kit II (Miltenyi Biotec) were adoptively transferred individually or in tandem into CD45.1 mice as previously described (25, 26, 37). After 24 hours, each mouse was primed intramuscularly (i.m.) with 15 μg of eOD-GT8 60mer or eOD-GT8-KO 60mer protein in 100 μl of PBS plus 2% Alhydrogel (Invitrogen) or a different dose of eOD-GT8 60mer mRNA-LNP (0.6 μg–10 μg) in 100 μl of PBS. Details are provided in figure legends. B cell responses from combined popliteal, inguinal, and sacral lymph nodes as well as splenocytes were measured by flow cytometry on days 14 and 42 post prime for CLK19 adoptive transfer experiments or days 14 and 36 post prime for other priming experiments. Sera were isolated from CLK19 adoptive transferred mice on days 14, 42, and 72 post prime for ELISA experiments. Reagents mentioned here and below are later described in detail (table S3).

Boosters (g5- or g28-mRNA-LNP) were delivered i.m. on day 42 post prime. After day 36 post-boost in the g5 or g28 experiments, popliteal, inguinal and sacral lymph nodes were isolated and combined for flow cytometry, whereas sera were isolated for ELISA experiments. In the g28v2 experiment, the same samples were taken on days 8, 16, and 28 post boost for flow cytometry and ELISA experiments as described above.

### Protein production

His-tagged and His-Avi-tagged monomeric antigens were produced as previously described (11). Nanoparticle 60mer immunogens were produced by transient transfection of HEK-293F cells (ThermoFisher) and purified by *Galanthus nivalis* lectin affinity chromatography (Vectorlabs) followed by SEC using a Superose 6 16/600 PG column (Cytiva). Immunogen preps confirmed to contain <5 EU/mg of endotoxin using an Endosafe instrument (Charles River).

Genes encoding the antibody Fv regions were synthesized by GenScript and cloned into antibody expression vectors pCW-CHIg-hG1 and pCW-CLIg-hk. Monoclonal antibodies were produced using transient transfection of HEK-293F cells (ThermoFisher) and purified using rProtein A Sepharose Fast Flow resin (Cytiva).

### Flow cytometry

To evaluate GC responses in adoptively transferred mice, inguinal, popliteal, and sacral lymph nodes as well as spleens were isolated and crushed in FACS buffer (2% FBS in PBS). Alexa Fluor 488- and Alexa Fluor 647-conjugated eOD-GT8/g5/g28/g28v2 tetramer probes were used for the detection of double-positive-priming or boosting immune response and Alexa Fluor 594 conjugated eOD-GT8/g5/g28/g28v2-KO probe was used for the exclusion of off-target binders. The staining procedure was performed with 1μg/ml of antibodies as previously reported (26).

To sort antigen-specific GC CD45.2 B cells for single-cell PCR, cells were incubated with 200-fold diluted B cell surface markers after staining with probes as described above. Single-cell plates were sorted by FACSAria-II SORP machine (BD) with a 100-μm nozzle and gated as lymphocytes^+^/singlets^+^/dump^−^(anti-mouse CD4, CD8, Ly-6G, F4/80)/Live-Dead^−^/B220^+^/CD95^+^CD38^−^/CD45.2^+^/Ag^+^ (AF488^+^ and AF647^+^)/IgM^−^IgD^−^/AgKO^−^(AF594^−^).

To sort sufficient cells for the competition analysis (Fig. 3 and fig. S2) in the priming experiment with eOD-GT8 60mer, antigen-specific CD45.2 B cells were sorted by both single-cell plate sorting as described above and bulk sorting with a 70-μm nozzle for 10x Genomics.

### Enzyme-linked immunosorbent assays (ELISAs)

To assess the IgG titers of immunized mice, 50 ng per well of either eOD-GT8, g5, g28, g28v2, or their respective KO equivalents, were precoated in 96-well plates at 4°C overnight. After incubation with blocking buffer (3% BSA in PBS + 0.01% tween 20) for 2 hours at RT, three-fold serial diluted sera (seven dilutions total) with starting dilution 1:100 from priming or boosting mice were incubated with precoated protein for 2 hours at RT, followed by incubation with 3000-fold diluted alkaline phosphatase AffiniPure goat anti-mouse IgG for 1 hour at RT. Finally, 50 μl per well of p-Nitrophenyl phosphate dissolved in ddH_2_O was added prior to an incubation for 20 min at RT for chromogenic reaction. The OD405 was read by ELISA readers (BioTek) as in (26).

### BCR sequencing

To assess antibody evolution and mutation frequency after priming or boosting, single-cell PCR was performed using sorted B cells in 96-well plates. Briefly, cDNA was prepared by RT-PCR with SuperScriptTM III Reverse Transcriptase kit (Thermo Fisher), and IgG and IgKappa were amplified by nested PCR as previously reported (77). Finally, Sanger sequencing was performed by GENEWIZ (Cambridge, MA). Sequences were quality-checked, aligned, and analyzed using the Geneious software (Biomatters Ltd, New Zealand). IMGT/V-QUEST (78–80) was used for Human Ig gene analysis

BCR sequencing for competition analysis in the priming experiment as shown in Fig. 3 and fig. S2 was performed by 10x Genomics. Briefly, lymph nodes from priming experiments were prepared for flow cytometry staining as described above. During staining, each sample was barcoded using 2 μl of anti-mouse hashtag antibody (Biolegend). NGS libraries were generated from approximately 7000 sorted B cells following the 10x Genomics Chromium Next GEM Single Cell 5′ Reagent Kits v2 protocol (10x Genomics). Average library size was determined using Agilent TapeStation Systems D5000 high sensitivity Screen Tape assay (Agilent, Santa Clara, CA). Libraries were quantified by Qubit dsDNA High Sensitivity (Invitrogen). Libraries were pooled and sequenced using a NextSeq 550 System (Illumina, San Diego, CA). Sequences were analyzed using a customized regions reference file and the Cell Ranger v.6 software pipeline (10x Genomics).

### Antibody affinity detection by surface plasmon resonance (SPR)

Kinetics and affinity of antibody–antigen interactions were measured on a Carterra LSA using the methods described in Cottrell *et al*. (56).

### Molecular modeling

A de novo model for core-g28v2 was generated using AlphaFold2 and Man5 glycans were added and relaxed using Rosetta. De novo models for e1–1_d16_G02 and e2–3_d30_F02 Fvs were generated with IgFold (81). Models were fit onto 4JPW in UCSF Chimera to create an initial model for docking (82, 83). Docking was performed using Rosetta Relax without starting coordinate restraints and including glycan specific scoring function terms (84). Docked models were scored using MolProbity and the final model for each Fv core-g28v2 pair was selected based on lowest MolProbity score (85). Figures were made using UCSF Chimera (83).

### Statistical analysis

Significant differences were calculated with Student’s *t* test (between two groups) or one-way-ANOVA (among three or more groups) and shown as: no significant difference *P*>0.05, *P<0.05, **P<0.01, ***P<0.001, ****P<0.0001. All *P*-value analyses were calculated using GraphPad Prism V8.0.

## Supplementary Material

Supplemental Data 1

Supplemental Data 2

Supplementary Material

## Data Availability

Model animals available from F.D.B. on request, under a standard material transfer agreement with the Massachusetts General Hospital. Codes used for computational modeling deposited to Zenodo (86). Sequences used for affinity measurement have been deposited in Genbank (accession numbers: OR441107–OR441306). All other sequence data have been deposited in Dryad (87). Plasmids or proteins for immunogens and sort reagents related to eOD-GT8 60mer, core-g5 60mer, core-g28 60mer, and core-g28v2 60mer, or antibodies CLK09, CLK19, and CLK21, or SPR reagents in this study, are available from W.R.S. under a material transfer agreement with The Scripps Research Institute. mRNA-LNP vaccine constructs can be made available from S.H. under a material transfer agreement with Moderna. All other data are available in the main text or the supplementary materials including two supplemental data files: data S1 (related to Figs. 4F, 5G, and 7F) and data S2 (related to Fig. 7D).
