# Supplementary material for: mRNA-LNP prime–boost evolves precursors toward VRC01-like broadly neutralizing antibodies in preclinical humanized mouse models
Source: Sci Immunol. Author manuscript; Available in PMC 2024 Oct 18. (PMC11488661; doi:10.1126/sciimmunol.adn0622)
Supplement: Supplementary Material [file NIHMS2022938-supplement-Supplementary_Material.pdf]

Supplementary Materials for

mRNA-LNP prime–boost evolves precursors toward VRC01-like broadly neutralizing antibodies in preclinical humanized mouse models

Xuesong Wang, Christopher A. Cottrell, Xiaozhen Hu, Rashmi Ray, Maria Bottermann, Paula Maldonado Villavicencio, Yu Yan, Zhenfei Xie, John E. Warner, Jordan Renae Ellis-Pugh, Oleksandr Kalyuzhniy, Alessia Liguori, Jordan R. Willis, Sergey Menis, Sebastian Rämisch, Saman Eskandarzadeh, Michael Kubitz, Ryan Tingle, Nicole Phelps, Bettina Groschel, Sunny Himansu, Andrea Carfi, Kathrin H. Kirsch, Stephanie R. Weldon, Usha Nair, William R. Schief, and Facundo D. Batista

Corresponding authors: schief@scripps.edu (W.R.S) and fbatista1@mgh.harvard.edu (F.D.B.)

The PDF file includes:

Figs. S1 to S8
Tables S1 to S3
Legends for Data S1 and S2

References (89, 90)

Other Supplementary Materials for this manuscript include the following:

Data S1 and S2

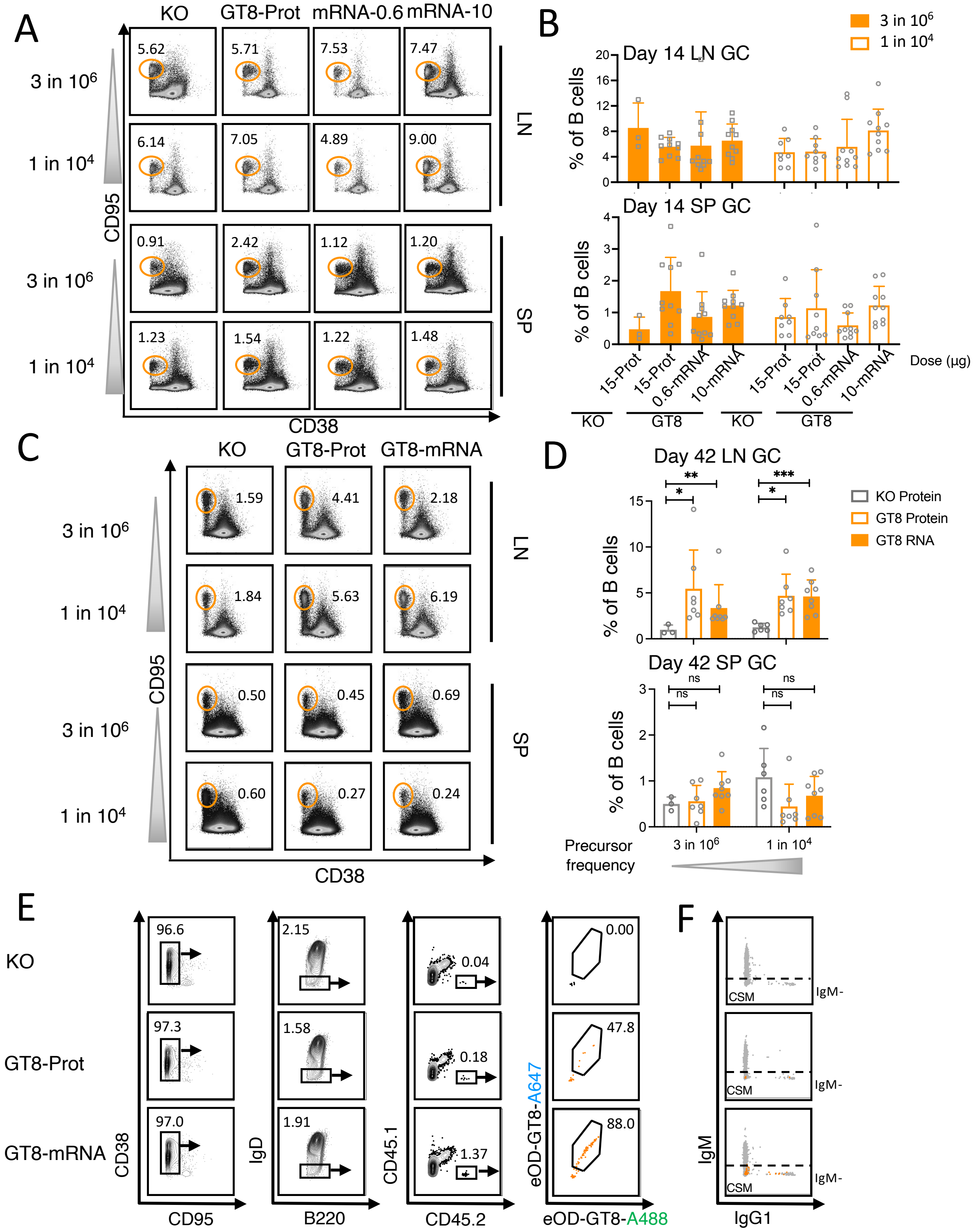

Fig. S1. B cell immune responses primed by protein and eOD-GT8 60mer mRNA-LNP in CLK19 mice with variable precursor frequencies, related to Fig. 1. Mice adoptively transferred

with CLK19 were generated with varying precursor frequencies and primed with eOD-GT8 60mer mRNA, as described in Fig. 1A. **(A)** Flow cytometry of GC B cells at day 14. The lymph nodes (LN) (top two rows) and splenocytes (SP) (bottom two rows) were isolated from cohorts immunized with eOD-GT8 60mer/KO protein or different doses of eOD-GT8 60mer mRNA-LNP for analysis. GC B cells were detected by flow cytometry with the marker $SSL^{+}/B220^{+}/CD95^{+}CD38^{-}$. Gated plots represent the percentage of GC B cells. (**B**) Quantification of GC B cells from lymph nodes (upper) or spleen (lower) of mice adoptively transferred and immunized as in Fig. 1A. Two independent experiments with 3–5 mice per group were analyzed. Bars indicate geometric means and geometric SD from pooled groups. (**C**) As in (A), but on day 42. (**D**) As in (B), but on day 42. Two independently repeated experiments with 3–4 mice per group were analyzed. Bars indicate geometric means and geometric SD from pooled groups. One-way ANOVA was used for significance analysis. ns $P>0.05$, $^{*}P<0.05$, $^{**}P<0.01$, $^{***}P<0.001$, . (**E**) Flow cytometry of the memory B cell (MBC) response induced by eOD-GT8 60mer protein or mRNA-LNP in CLK19 adoptively transferred mice (1-in-10^4 precursor frequency) on day 42. Panels were prior gated from $SSL^{+}B220^{+}$. (**F**) Class switched memory B cells (CSM) were gated as $SSL^{+}B220^{+}CD38^{+}CD95^{-}IgD^{lo/-}IgM^{-}$. Orange dots represent eOD-GT8-specific CD45.2 CSM.

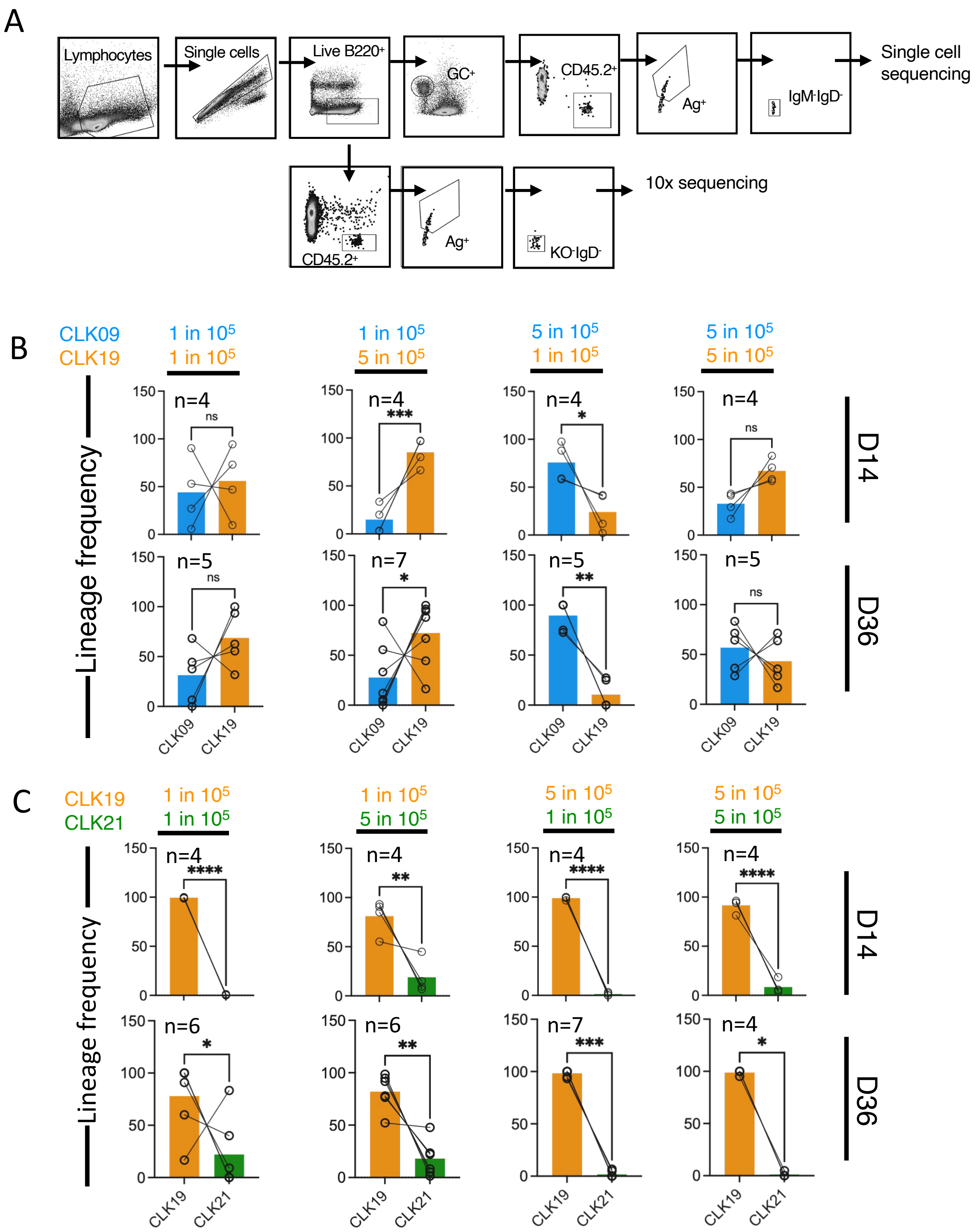

A
Lymphocytes
Single cells
Live B220+
GC+
CD45.2+
Ag+
IgM-IgD-
Single cell sequencing
CD45.2+
Ag+
KO-IgD-
10x sequencing
B
CLK09
CLK19
1 in 10^5
5 in 10^5
Lineage frequency
n=4
n=5
n=7
ns
*
**
***
D14
D36
CLK09
CLK19
C
CLK19
CLK21
n=6
****

Fig. S2. Sequence frequency in individual mice bearing two types of CLK BCRs after eOD-GT8 60mer mRNA-LNP priming, related to Fig. 3. Mice adoptively transferred with two types of CLK KI B cells at variable starting ratios were generated and primed with eOD-GT8 mRNA-LNP as described in Fig. 3A. (**A**) Gating strategy of sorted B cells. Lymph nodes from mRNA-LNP immunized mice were isolated at 14 and 36 days and sorted as shown, $IgM^{-}IgD^{-}$ eOD-GT8-specific CD45.2 within GC were sorted for single-cell-plate sequencing for human IgG and IgKappa; $KO^{-}IgD^{-}$ eOD-GT8 specific CD45.2 from total B cells were sorted for 10x sequencing for human IgG and IgKappa. (**B**) Sequence frequency of CLK lineages after priming in CLK09-19. Circles represent the frequency of CLK09 (blue) or CLK19 (orange) within one mouse. Connecting lines indicate CLK09 and CLK19 sequence frequency from the same mouse. n=2–4 mice per group for each independent experiment, and two independent experiments were analyzed. Bars indicate geometric means and geometric SD from pooled groups. Significance was calculated with Student's *t* test and shown as: ns $P>0.05$, *$P<0.05$, **$P<0.01$, ***$P<0.001$. (**C**) As in (B), but for CLK19-21 with CLK19 (orange) and CLK21 (green).

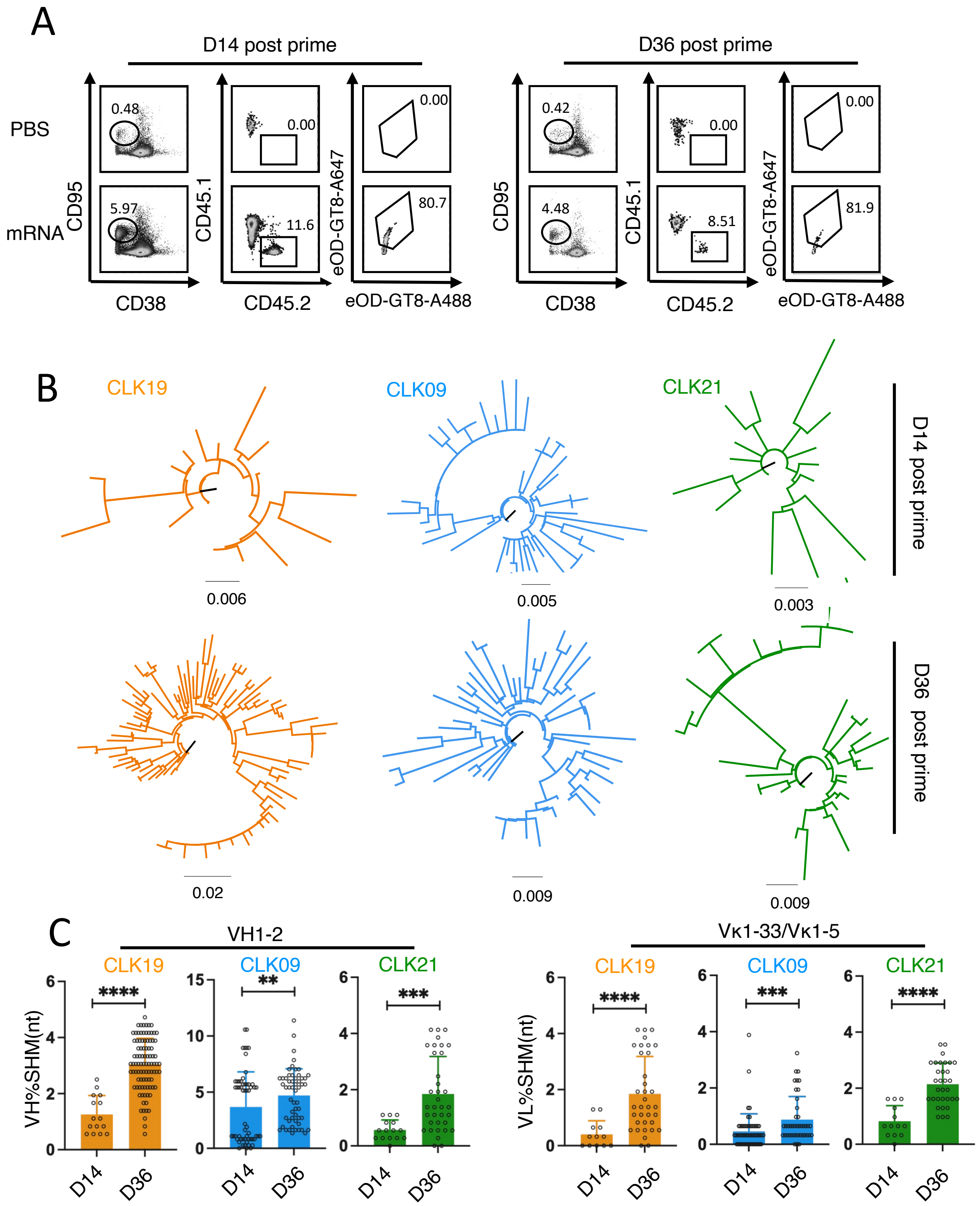

Fig. S3. GC responses induced by eOD-GT8 60mer mRNA-LNP in CLK mice bearing human mini-B cell repertoire, related to Fig. 4. Mice bearing mini-B cell repertoires were generated and

primed with eOD-GT8 mRNA-LNP as described in Fig. 4A. (**A**) Flow cytometry of GC response induced by eOD-GT8 mRNA-LNP over time. The lymph nodes were isolated at day 14 (left) and 36 (right), and the CD45.2 binders were detected using eOD-GT8 probes by flow cytometry FACS with the marker $SSL^{+}/B220^{+}/CD95^{+}CD38^{-}/CD45.2^{+}/Ag^{+}$. (**B**) Phylogenetic tree of CLK lineages over time. These trees were generated from paired CLK19 (orange), CLK09 (blue), and CLK21 (green) sequences of HC and LC from human-mini-B cell repertoire induced by eOD-GT8 60mer mRNA-LNP at day 14 (upper) and 36 (lower). CLK paired nt sequences were joined and aligned by MUSCLE (*89*) and trees were generated using FastTree and a Jukes–Cantor model (*90*) for nt evolution. The branch length reflects the sequence distance. (**C**) Percent nt change in IGHV (left) and IGLV (right) of CLK19, CLK09, and CLK21 lineages isolated from GC at day 14 and 36. Each dot represents one sequence from CLK19 (orange), CLK09 (blue), and CLK21 (green) lineages. Data from two independent experiments were analyzed. Each dot represents one sequence. Bars indicate geometric means and geometric SD of 12–63 sequences per group from pooled experiments. Significance was calculated with Student's *t* test and shown as: $**P<0.01$, $***P<0.001$, $****<0.0001$.

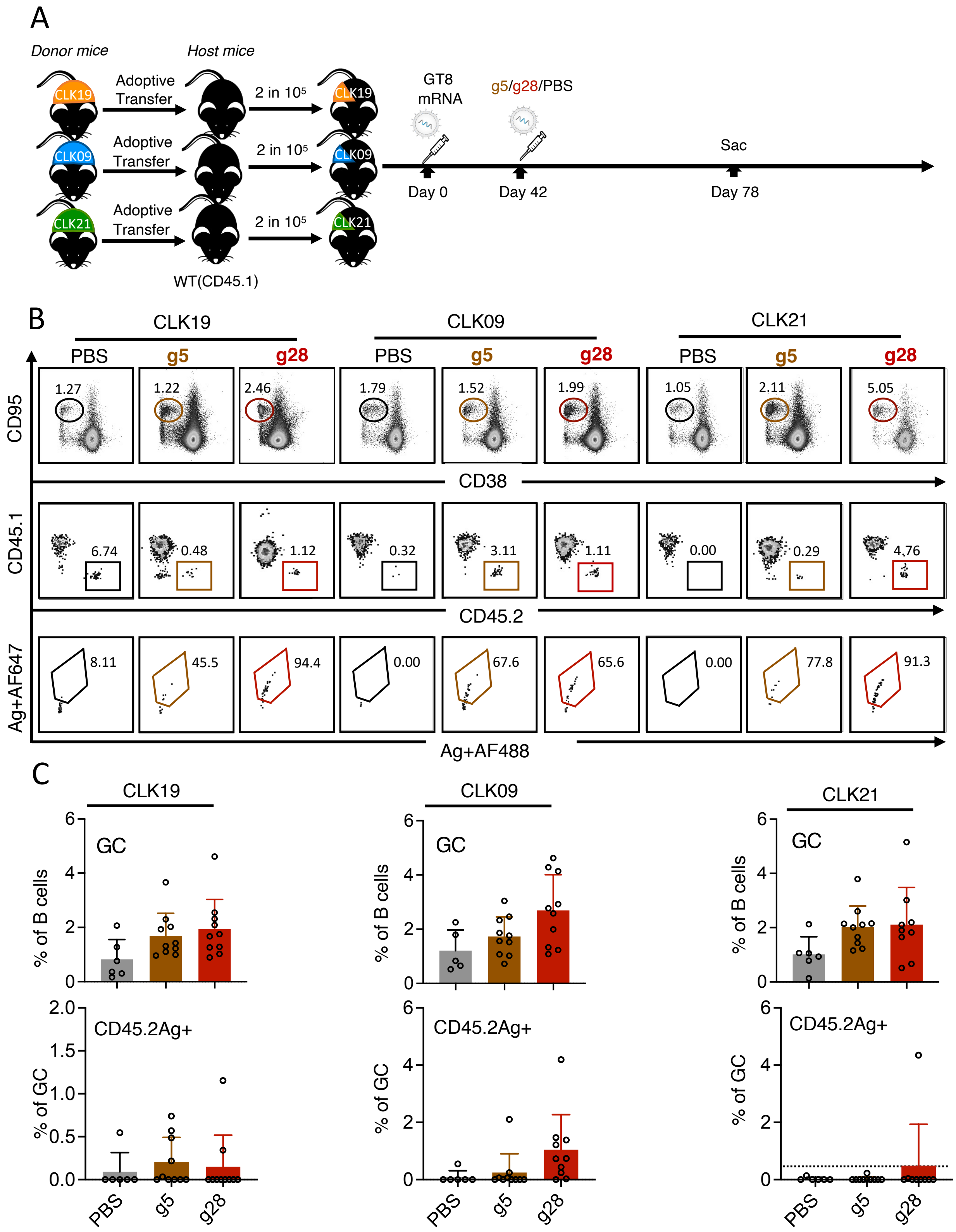
A
Donor mice
Host mice
CLK19
CLK09
CLK21
Adoptive Transfer
2 in 10⁵
WT(CD45.1)
GT8 mRNA
g5/g28/PBS
Sac
Day 0
Day 42
Day 78
B
CLK19
CLK09
CLK21
PBS
g5
g28
CD95
CD38
CD45.1
CD45.2
Ag+AF647
Ag+AF488
1.27
1.22
2.46
1.79
1.52
1.99
1.05
2.11
5.05
6.74
0.48
1.12
0.32
3.11
1.11
0.00
0.29
4.76
8.11
45.5
94.4
0.00
67.6
65.6
0.00
77.8
91.3
C
GC
% of B cells
CD45.2Ag+
% of GC
PBS
g5
g28

Fig. S4. Prime–boost regimens using g5 or g28 induce activation in recipients of single CLK lineage, related to Fig. 5. (**A**) Schematic of single adoptive transfers and prime–boost immunization. (**B**) Flow cytometry of GC response induced after GT8-g5 or GT8-g28 prime–boost mRNA-LNP regimen on day 78. A probe including both g5 and g28 was used to detect CD45.2 binders from the GT8-prime group. A probe of g5 or g28 alone was used to detect CD45.2 binders from GT8-g5-prime–boost group or GT8-g28-prime–boost group, respectively. CD45.2 Ag^{+} binder populations were gated with the marker $SSL^{+}/B220^{+}/CD95^{+}CD38^{-}/CD45.2^{+}/Ag^{+}$. Gated plots represent the percentage of GC (upper row), CD45.2 (middle row) and Ag^{+}CD45.2 (bottom row). (**C**) Quantification of Ag^{+}CD45.2 within GCs induced by prime–boost strategy. The graphs show GC B cells (upper) and GC CD45.2 binders (lower) at day 78. The *x*-axis represents the immunization group. The *y*-axis represents the percentage of GC B cells (upper) and GC CD45.2 binders (lower). Each circle represents one mouse. Two independent experiments with 3–5 mice per independent group were analyzed. Bars indicate geometric mean and geometric SD from mice in pooled groups.

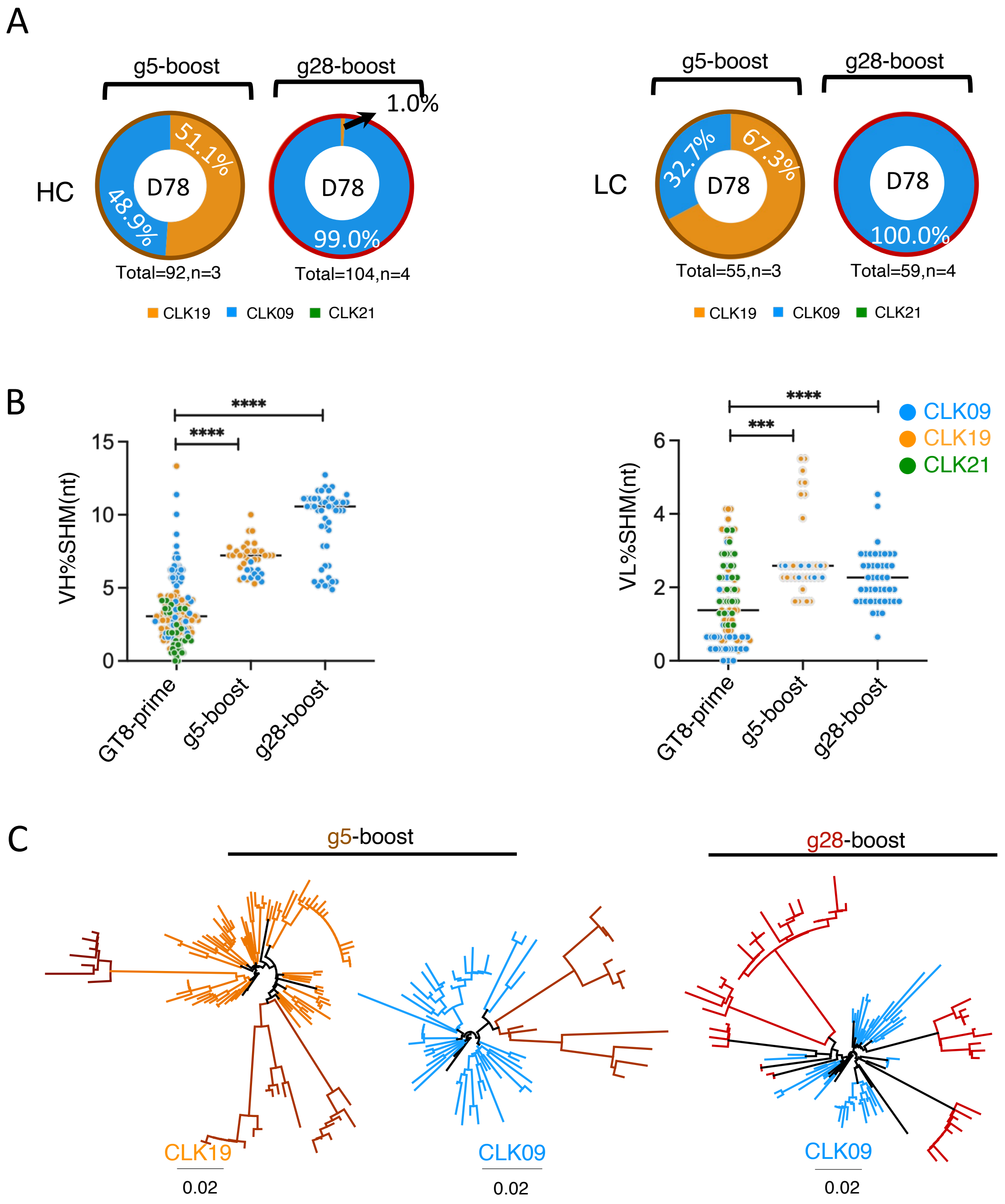

A
g5-boost
g28-boost
HC
51.1%
48.9%
D78
1.0%
99.0%
D78
Total=92,n=3
Total=104,n=4
CLK19 CLK09 CLK21
g5-boost
g28-boost
LC
32.7%
67.3%
D78
D78
100.0%
Total=55,n=3
Total=59,n=4
CLK19 CLK09 CLK21
B
VH%SHM(nt)
****
****
GT8-prime
g5-boost
g28-boost
VL%SHM(nt)
****
***
GT8-prime
g5-boost
g28-boost
CLK09
CLK19
CLK21
C
g5-boost
g28-boost
CLK19
0.02
CLK09
0.02
CLK09
0.02

Fig. S5. SHM after g5 and g28 prime–boost in mini-repertoire mice, related to Fig. 5. (**A**) Sequence frequency of CLK lineages in GC after g5 or g28 boosting. Charts show frequencies of CLK09 (blue), CLK19 (orange), and CLK21 (green) lineages for HC (left) and LC (right) from g5-or g28-boosting group. Total: sequences per group, n: mice per group. (**B**) Percent SHM, in nucleotides, in IGHV (left) and IGLV (right) of CLK19 (orange), CLK09 (blue) and CLK21 (green) lineages from GT8-prime (day 36 prime-only sequence data presented initially in Fig. 4C repeated here for comparison)- and g5-or g28-boost-groups. Each dot represents one sequence, 37–130 sequences per group from two independent experiments pooled for analysis. Significance was calculated with one-way-ANOVA: ***P<0.001, ****P<0.0001. (**C**) Phylogenetic trees of CLK lineages over time, with post-boost (brown or red) lines overlayed on post-prime trees. These trees were generated from paired CLK19 (orange), CLK09 (blue) and CLK21 (green) sequences from human-mini-B cell repertoire primed by eOD-GT8 60mer mRNA-LNP at day 36 (day 36 prime-only sequence data presented initially in Fig. 4C repeated here for comparison). Phylogenetic trees of CLK lineages post boost were generated using FastTree and a Jukes–Cantor model as mentioned in fig. S3B. The branch lengths reflect the sequence distance.

A

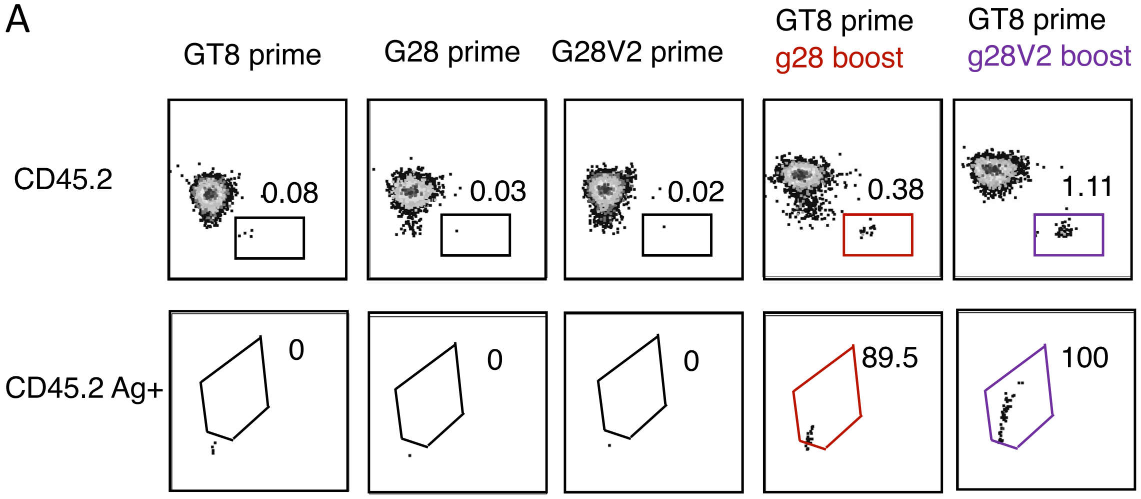
GT8 prime
G28 prime
G28V2 prime
GT8 prime
g28 boost
GT8 prime
g28V2 boost
CD45.2
0.08
0.03
0.02
0.38
1.11
CD45.2 Ag+
0
0
0
89.5
100

B

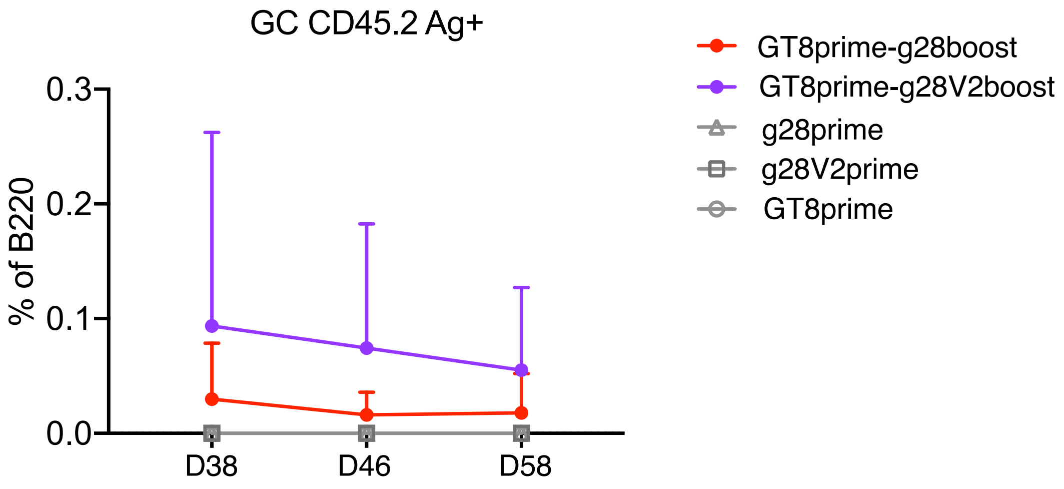
GC CD45.2 Ag+
% of B220
0.3
0.2
0.1
0.0
D38
D46
D58
GT8prime-g28boost
GT8prime-g28V2boost
g28prime
g28V2prime
GT8prime

Fig. S6. Comparison of GC response induced by g28 and g28v2 boosters, related to Fig. 6. (**A**) Flow cytometry of CD45.2 binders within GCs after prime–boost immunization. The g28 or g28v2 probe was used to detect CD45.2 binders, which were then gated with the markers $SSL^+/B220^+/CD95^+CD38^-/CD45.2^+/Ag^+$. Plots show the percentage of CD45.2 (upper row) and antigen specific CD45.2 B cells (bottom row) out of GC. (**B**) Kinetics of GC CD45.2 binders over time after g28 (red) or g28v2 (purple) boosting. The *x*-axis represents the time points for boosting and the *y*-axis represents the percentage of GC CD45.2 binders out of B cells. Two independently repeated experiments with 3–5 mice per independent group were analyzed. Each circle or square with bars indicates geometric means and geometric SD from mice in pooled groups.

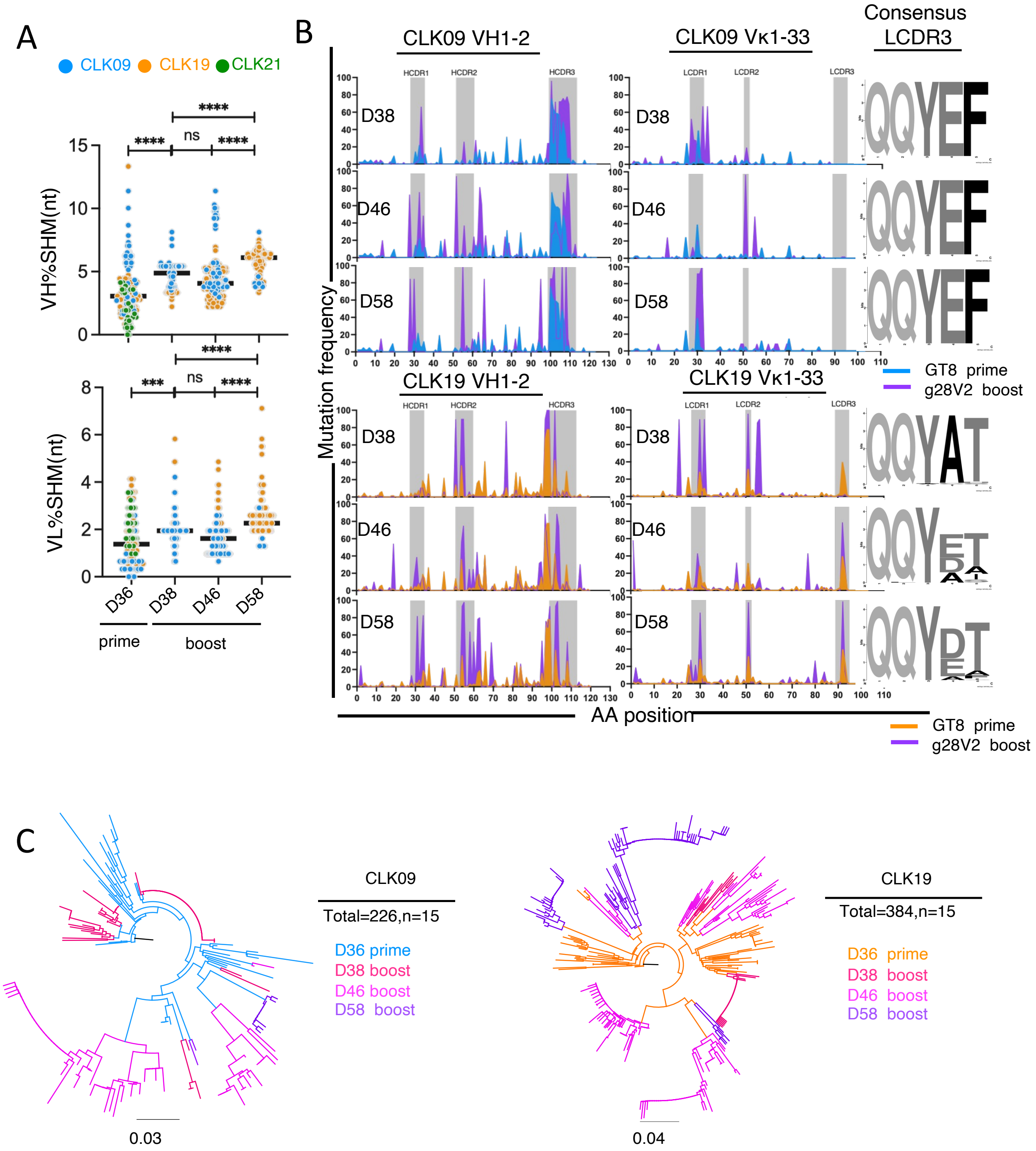

A
CLK09 CLK19 CLK21
VH%SHM(nt)
VL%SHM(nt)
D36 D38 D46 D58
prime
boost
B
CLK09 VH1-2
CLK09 Vκ1-33
Consensus LCDR3
CLK19 VH1-2
CLK19 Vκ1-33
Mutation frequency
AA position
GT8 prime
g28V2 boost
C
CLK09
Total=226,n=15
D36 prime
D38 boost
D46 boost
D58 boost
CLK19
Total=384,n=15
D36 prime
D38 boost
D46 boost
D58 boost
0.03
0.04

Fig. S7. SHM after delivery of boost immunogen g28v2, related to Fig. 7. (**A**) Percent SHM, in nucleotides, in IGHV (upper) and IGLV (lower) of CLK19 (orange), CLK09 (blue), and CLK21 (green) lineages from GT8-prime-and g28v2-boost groups. Each dot represents one sequence, 118–240 sequences per group from two independent experiments were pooled for analysis. Significance was calculated with one-way-ANOVA: ns $p>0.05$, ***$P<0.001$, ****$P<0.0001$. (**B**) AA mutation distribution was assessed via hotspot analysis for VH1-2 (left) and Vκ1-33 (right) in CLK09 (upper, prime-only blue) and CLK19 (lower, prime-only orange). Post-boost frequencies overlaid in purple. WebLogos at right (*88*). (**C**) Phylogenetic tree of CLK lineages over time, with post-boost (shades of pink and violet, marking change over time) lines overlayed on 36 days post-prime trees. These trees were generated from paired CLK19 (prime-only: orange) and CLK09 (prime-only: blue) with models as described in fig. S3B. The branch length reflects the sequence distance. All sequences after prime are calculated from data at 36 days post-prime first shown in Fig. 4C.

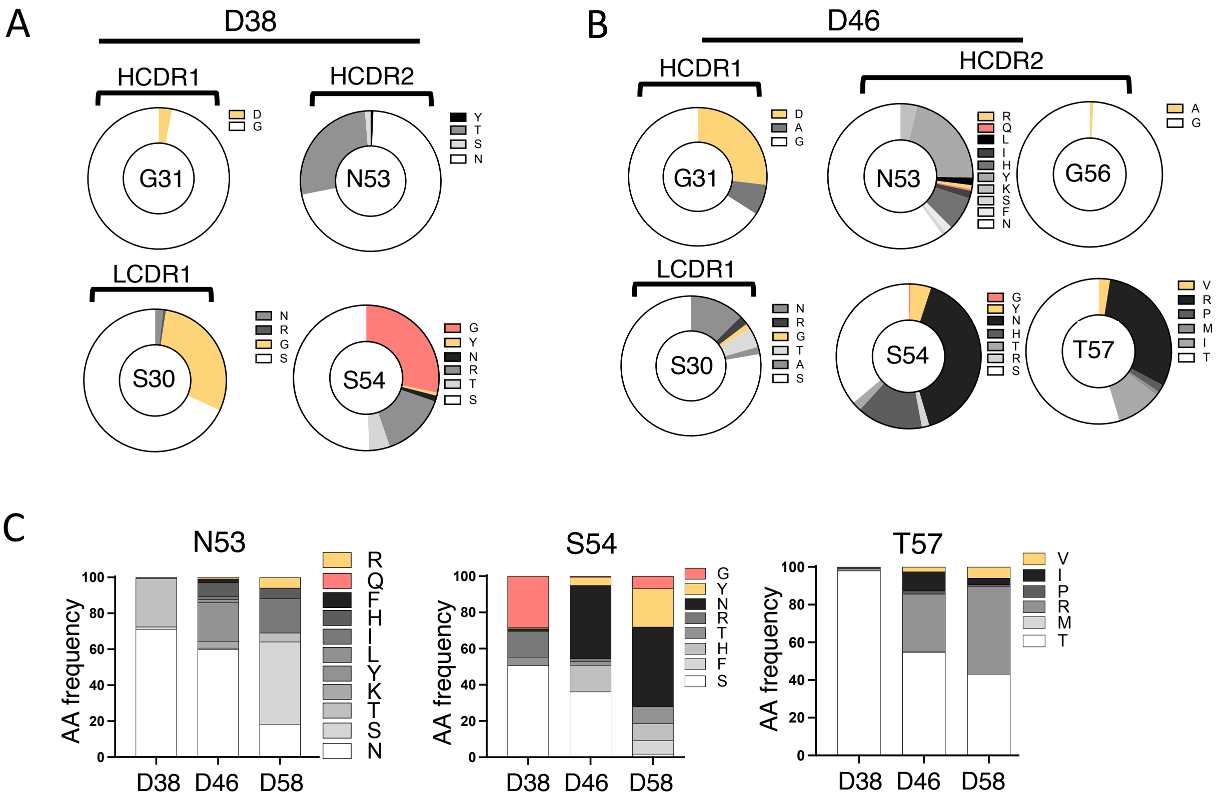

Fig. S8. Key mutations after immunization by g28v2, related to Fig. 7. (**A** and **B**) Frequency of key CDR mutations at (A) 8 days post-boost (D38) and (B) 16 days post-boost (D46) from both CLK09 and CLK19. Key residues (determined in reference to the mature VRC01-class bnAbs) were analyzed as in Fig. 7C. (**C**) Mutation accumulation over time. The *x*-axis represents the time points and the *y*-axis represents the frequency of mutated aa in key residues of HCDR2, including N53, S54, and T57.

Table S1. SHM frequency for CLK lineages after eOD-GT8 60mer mRNA-LNP priming.

Timepoint	D14			D36		
**Mean of aa SHMs (%)**	CLK19	CLK09	CLK21	CLK19	CLK09	CLK21
**HC**	2.9	4.2	1.1	6.5	6.7	3.2
**LC**	0.8	0.9	1.4	2.4	1.5	4.2
**Mean of nt SHMs (%)**	CLK19	CLK09	CLK21	CLK19	CLK09	CLK21
**HC**	1.3	3.7	0.6	3.0	4.7	1.8
**LC**	0.4	0.5	0.8	1.8	0.9	2.1

Means of amino acid (aa) and nucleotide (nt) SHM frequency shown in the table are related to Fig. 4D and Fig. S3C.

Table S2. Key mutations in heavy chain over time after g28v2 boosting.

AA Residue #.	19	30	31	32	33	34	51	53	54	56	57	61	81	82a
D38	-	-	D	-	F	I	-	T	R/G/Y/N/T	-	-	-	-	R
D46	R	I	D/A	H	F	I/L	V/L	K/L/R/Q	R/G/Y/N/T	A	P/R/V	R/H	D	R
D58	R	-	D	H	F	I/L	-	R/T	Y/F/G/N/T	A	P/R/V	R	-	-

Light gray indicates a key mutation position in HCDR1, while dark gray indicates a key mutation position in HCDR2.

Table S3. Key resources, related to the Materials and Methods.

REAGENT or RESOURCE	SOURCE	IDENTIFIER
**Antibodies**		
PerCP/Cy5.5 anti-mouse CD45.1 Antibody	Biolegend	Cat#: 110728
PE-Cy™7 Hamster Anti-Mouse CD95	BD Biosciences	Cat#: 557653
Brilliant Violet 785™ anti-mouse CD45.2	Biolegend	Cat#: 109839
anti-mouse CD38, BV510	BD Biosciences	Cat#: 740129
Pacific Blue™ anti-mouse/human CD45R/B220 Antibody	Biolegend	Cat#: 103227
BV711 Rat Anti-Mouse Ig, λ1, λ2 & λ3 Light Chain	BD Biosciences	Cat#: 744527

BUV395 Rat Anti-Mouse Ig, κ light chain	BD Biosciences	Cat#: 742839
APC/Cy7 anti-mouse/human CD45R/B220 Antibody	Biolegend	Cat#: 103224
BV421 Rat Anti-Mouse IgM	BD Biosciences	Cat#: 743323
PE/Cy7 anti-mouse IgD Antibody	Biolegend	Cat#: 405720
PE anti-mouse CD45.2 Antibody	Biolegend	Cat#: 109808
Anti-mouse CD38 Alexa700	Thermo Fisher Scientific	Cat#: 56-0381-82
BV786 Rat Anti-Mouse IgD	BD Biosciences	Cat#: 563618
BV510 Rat Anti-Mouse B220/CD45R Clone RA3-6B2	BD Biosciences	Cat#: 563103
BV421 Rat Anti-Mouse IgG1	BD Biosciences	Cat#: 562580
BUV395 Rat Anti-Mouse IgM	BD Biosciences	Cat#: 743329
BUV395 Rat Anti-Mouse CD273	BD Biosciences	Cat#: 565102
CD4 Monoclonal Antibody (GK1.5), APC-eFluor 780	Thermo Fisher Scientific	Cat#: 47-0042-80
CD8a Monoclonal Antibody (53-6.7), APC-eFluor 780	I Thermo Fisher Scientific	Cat#: 47-0081-80
F4/80 Monoclonal Antibody (BM8), APC-eFluor 780	Thermo Fisher Scientific	Cat#: 47-4801-80
Ly-6G Monoclonal Antibody (1A8-Ly6g), APC-eFluor 780	Thermo Fisher Scientific	Cat#: 47-5931-80
Alexa Fluor® 488 anti-mouse CD38 Antibody	Biolegend	Cat#: 102714
Alexa Fluor® 594 anti-mouse/human CD45R/B220 Antibody	Biolegend	Cat#: 103254
Alkaline Phosphatase AffiniPure Goat Anti-Mouse IgG, Fcγ Fragment Specific	Jackson ImmunoResearch	Cat#: 115-055-071
Purified Rat Anti-Mouse CD16/CD32 (Mouse BD Fc Block™	BD Biosciences	Cat#: 553142
Alexa Fluor® 488 anti-mouse CD45.2 Antibody	Biolegend	Cat#: 109816
Alexa Fluor® 594 anti-mouse CD3 Antibody	Biolegend	Cat#: 100240
TotalSeq™-C0301 anti-mouse Hashtag 1 Antibody	Biolegend	Cat#: 155861
TotalSeq™-C0302 anti-mouse Hashtag 2 Antibody	Biolegend	Cat#: 155863
TotalSeq™-C0303 anti-mouse Hashtag 3 Antibody	Biolegend	Cat#: 155865
TotalSeq™-C0304 anti-mouse Hashtag 4 Antibody	Biolegend	Cat#: 155867
TotalSeq™-C0305 anti-mouse Hashtag 5 Antibody	Biolegend	Cat#: 155869
TotalSeq™-C0306 anti-mouse Hashtag 6 Antibody	Biolegend	Cat#: 155871
TotalSeq™-C0307 anti-mouse Hashtag 7 Antibody	Biolegend	Cat#: 155873
TotalSeq™-C0308 anti-mouse Hashtag 8 Antibody	Biolegend	Cat#: 155875

TotalSeq™-C0309 anti-mouse Hashtag 9 Antibody	Biolegend	Cat#: 155877
TotalSeq™-C0310 anti-mouse Hashtag 10 Antibody	Biolegend	Cat#: 155879
**Chemicals, Peptides, and Recombinant Proteins**		
LIVE/DEAD™ Fixable Blue Dead Cell Stain Kit, for UV excitation	Thermo Fisher Scientific	Cat#: L34962
Invitrogen™ Molecular Probes™ DAPI (4',6-Diamidino-2-Phenylindole, Dihydrochloride)	Thermo Fisher Scientific	Cat#: D1306
Chromium Next GEM Single Cell 5′ Kit v2	10x Genomics	PN-1000263
Library Construction Kit	10x Genomics	PN-1000190
Chromium Single Cell Mouse BCR Amplification Kit	10x Genomics	PN-1000255
Chromium Next GEM Chip K Single Cell Kit	10x Genomics	PN-1000286
Dual Index Kit TT Set A	10x Genomics	PN-1000215
Dual Index Kit TN Set A	10x Genomics	PN-1000250
Alexa Fluor 488 Streptavidin	Biolegend	Cat#: 405235
Alexa Fluor 647 Streptavidin	Biolegend	Cat#: 405237
Alexa Fluor 594 Streptavidin	Biolegend	Cat#: 405240
Pan B Cell Isolation Kit II, mouse	Miltenyi Biotec	Cat#: 130-104-443
SuperScript™ III Reverse Transcriptase	Thermo Fisher	Cat#: 18080085
HotStarTaq DNA Polymerase	Qiagen	Cat#: 203205
RNasin® Ribonuclease Inhibitors (Recombinant)	Promega	Cat#: N2515
CountBright™ Absolute Counting Beads, for flow cytometry	Thermo Fisher Scientific	Cat#: C36950
SIGMAFAST™ p-Nitrophenyl phosphate Tablets	Sigma	Cat#: N2770-50SET
NP40	Millipore	Cat#: 492016-100ML
UltraComp eBeads™ Compensation Beads	Thermo Fisher Scientific	01-2222-42
eOD-GT8 60mer	(*18*)	N/A
eOD-GT8-KO 60mer	(*18*)	N/A
eOD-GT8 His-tagged soluble protein	(*18*)	N/A
eOD-GT8-KO His-tagged soluble protein	(*18*)	N/A
eOD-GT8 60mer mRNA	Moderna	N/A
core-g28 60mer mRNA	Moderna	N/A
core-g28v2 60mer mRNA	Moderna	N/A
core-g5 60mer mRNA	Moderna	N/A
core-g28 His-tagged soluble protein	Schief lab	N/A
core-g5 His-tagged soluble protein	Schief lab	N/A
core-g28v2 His-tagged soluble protein	Schief lab	N/A
core-g28 His-Avi-tagged soluble protein	Schief lab	N/A
core-g5 His-Avi-tagged soluble protein	Schief lab	N/A
core-g28v2 His-Avi-tagged soluble protein	Schief lab	N/A
BirA	Avidity	Bulk BirA
**Experimental Models: Organisms/Strains**		

Mouse: B6.SJL-Ptprcapepcb/BoyJ	The Jackson Laboratory	JAX: 002014
Mouse: CLK21 BCR KI mouse model	(*26*)	N/A
Mouse: CLK09 BCR KI mouse model	(*26*)	N/A
Mouse: CLK19 BCR KI mouse model	(*26*)	N/A
**Oligonucleotides**		
Primers for single cell PCR	GENEWIZ	N/A
**Software and Algorithms**		
Flowjo X	Treestar	https://www.flowjo.com/
Prism 8	GraphPad	https://www.graphpad.com/
Microsoft Office	Microsoft	https://www.office.com/
IMGT/HighV-QUEST	(*78–80*)	http://www.imgt.org/IMGTindex/V-QUEST.php
Geneious Prime	Biomatters	https://www.geneious.com/
UCSF Chimera	UCSF	https://www.cgl.ucsf.edu/chimera/
Alphafold2	Deepmind	https://github.com/deepmind/alphafold
Rosetta	University of Washington	https://www.rosettacommons.org
IgFold	Gray Lab (*81*)	https://colab.research.google.com/github/Graylab/IgFold/blob/main/IgFold.ipynb
MolProbity	Duke University (*85*)	http://molprobity.biochem.duke.edu/
Kinetics Data Analysis Software	Carterra	https://carterra-bio.com/applications/kinetics-software/

Data S1. KD values relating to the affinity data post prime-boost

Data S2. Key mutation values in heavy chain after priming or g28v2 boosting
